# Asporin-deficient mice have tougher skin and altered skin glycosaminoglycan content and structure

**DOI:** 10.1371/journal.pone.0184028

**Published:** 2017-08-31

**Authors:** Marco Maccarana, René B. Svensson, Anki Knutsson, Antonis Giannopoulos, Mea Pelkonen, MaryAnn Weis, David Eyre, Matthew Warman, Sebastian Kalamajski

**Affiliations:** 1 Department of Experimental Medical Sciences, Lund University, Lund, Sweden; 2 Institute of Sports Medicine, Bispebjerg Hospital, and Center for Healthy Aging, University of Copenhagen, Copenhagen, Denmark; 3 Department of Orthopaedics and Sports Medicine, University of Washington, Seattle, Washington, United States of America; 4 Children’s Hospital Boston, Howard Hughes Medical Institute, Harvard Medical School, Boston, Massachusetts, United States of America; 5 Department of Medical Biochemistry and Microbiology, Uppsala University, Uppsala, Sweden; Massey University, NEW ZEALAND

## Abstract

The main structural component of connective tissues is fibrillar, cross-linked collagen whose fibrillogenesis can be modulated by Small Leucine-Rich Proteins/Proteoglycans (SLRPs). Not all SLRPs’ effects on collagen and extracellular matrix *in vivo* have been elucidated; one of the less investigated SLRPs is asporin. Here we describe the successful generation of an *Aspn*^*-/-*^ mouse model and the investigation of the *Aspn*^*-/-*^ skin phenotype. Functionally, *Aspn*^-/-^ mice had an increased skin mechanical toughness, although there were no structural changes present on histology or immunohistochemistry. Electron microscopy analyses showed 7% thinner collagen fibrils in *Aspn*^-/-^ mice (not statistically significant). Several matrix genes were upregulated, including collagens (*Col1a1*, *Col1a2*, *Col3a1*), matrix metalloproteinases (*Mmp2*, *Mmp3*) and lysyl oxidases (*Lox*, *Loxl2*), while lysyl hydroxylase (*Plod2*) was downregulated. Intriguingly no differences were observed in collagen protein content or in collagen cross-linking-related lysine oxidation or hydroxylation. The glycosaminoglycan content and structure in *Aspn*^*-/-*^ skin was profoundly altered: chondroitin/dermatan sulfate was more than doubled and had an altered composition, while heparan sulfate was halved and had a decreased sulfation. Also, decorin and biglycan were doubled in *Aspn*^*-/-*^ skin. Overall, asporin deficiency changes skin glycosaminoglycan composition, and decorin and biglycan content, which may explain the changes in skin mechanical properties.

## Introduction

The bulk of the extracellular matrix (ECM) is composed of collagens woven into a fibrillar network that accommodates water-retaining proteoglycans and provides a cellular niche. Collagen fibrillogenesis and cross-linking can be modulated by Small Leucine-Rich Proteins/Proteoglycans (SLRPs); this constitutes a mechanism that contributes to the mechanical and physiological tissue properties [1–3]. The effects of SLRPs on collagen fibrillogenesis are evident in the organ-specific phenotypes of the various SLRP knockout mice: lumican-deficient mice have fragile skin and opaque cornea [4–6], fibromodulin-deficient mice have abnormally cross-linked and ill-fused collagen fibrils in tendons [3, 7], decorin-deficient mice have fragile skin, weak tendons and lower lung airway resistance [8–10], keratocan-deficient mice have flattened cornea (mimicking human cornea plana disorder caused by *KERA* mutations) [11, 12], and biglycan-deficient mice have osteoporotic bones [13]. Compound SLRP knockout mice have even graver multi-organ abnormities with more pronounced collagen fibril phenotypes [14, 15].

Not all SLRP knockout mouse phenotypes have been reported, but the collective knowledge of SLRPs’ tissue-specific effects and collagen interaction redundancy [16–18] will aid in understanding the complex mechanisms underlying the shaping of extracellular matrices. In this paper, we present an initial report on the phenotype of asporin-deficient mice. Compared with other SLRPs, asporin (ASPN or PLAP1) lacks glycosaminoglycan (GAG) chains [19] and carries a polymorphic calcium-binding polyaspartate sequence [17, 20]. Asporin expression has been detected in dermis, perichondrium and periosteum, tendon, and eye sclera [21, 22]. Concluded from several *in* vitro studies, asporin function has been related to collagen fibrillogenesis and collagen mineralization [17, 23–25]. Asporin has also been suggested to modulate cellular response to FGF-2 [26], BMP-2 [24], and TGF-β [20]. Asporin expression is induced by TGF-β [21, 27] and suppressed by IL-1β and TNF-α [27]. It can interact and inhibit TGF-β [28] and consequently act as a tumor suppressor [29], but other studies propose asporin to be an invasion-promoting protein [30–32].

In this paper we hypothesized that asporin functions in formation of a structurally coherent extracellular matrix *in vivo*. To test this hypothesis we generated an asporin knockout mouse model (*Aspn*^*-/-*^). We focused on analyzing the effect of asporin deficiency on skin extracellular matrix, where asporin has been detected [21, 22] and where we, in the current study, observed a biomechanical phenotype. To elucidate the underlying cause of this phenotype we also analyzed skin collagen, proteoglycans and GAGs. This report contributes to the overall understanding of the unique functions of SLRPs in connective tissues.

## Materials and methods

### Targeted disruption of the mouse *Aspn* gene

**Fig 1:**
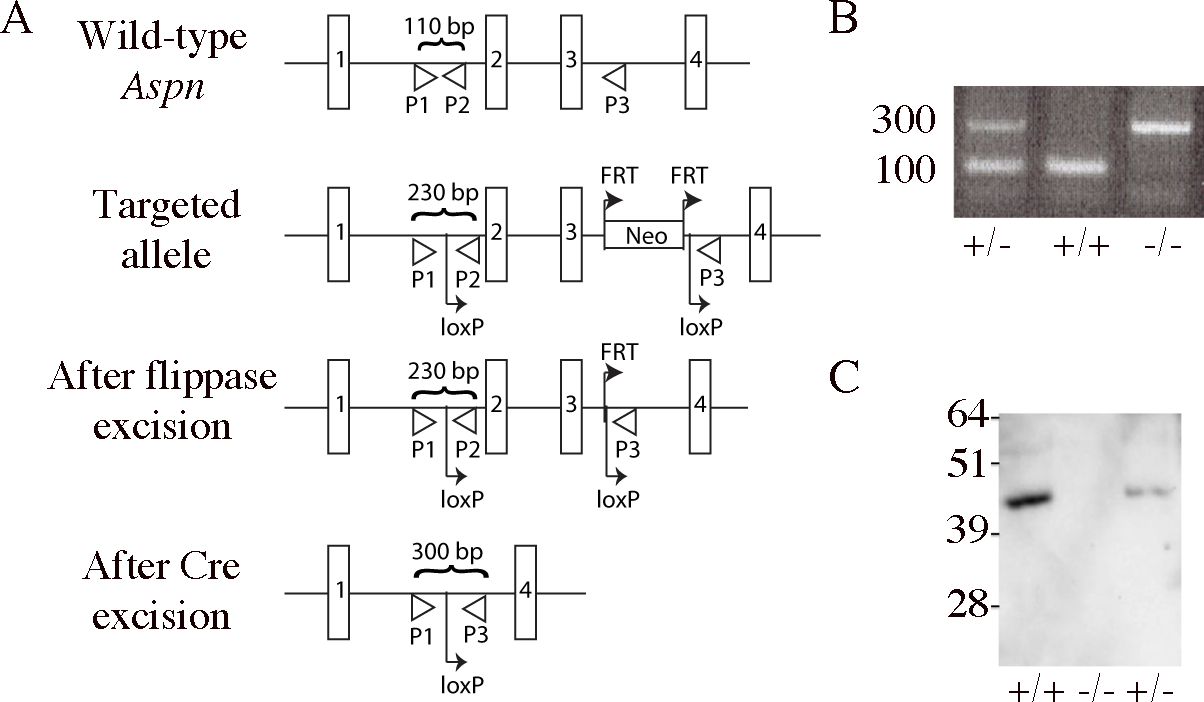
*Aspn*^*-/-*^ mouse generation. (A) Strategy for the creation of *Aspn-*null allele. The diagram shows wild-type, targeted, floxed, and excised alleles. Exons 1–4 (out of 8 present in the gene) are shown as rectangles. Primers used for genotyping are targeted to P1, P2, P3 sites and the expected PCR products’ sizes are denoted. (B) Genotyping PCR of *Aspn*^*-/-*^ mice using primers targeted to sites P1, P2, P3. (C) Confirmation of Aspn protein expression loss in *Aspn*^*-/-*^ mice by immunobloting tail SDS extracts for asporin.

All animal experiments were conducted under approval of the regional animal ethical board committee (Malmö-Lunds djurförsöksetiska nämnd, Jordbruksverket, Sweden). Mice were euthanized by carbon dioxide followed by cervical dislocation. The BAC clone bMQ103e19 containing the 129/Sv mouse *Aspn* genomic material was obtained from Geneservice (UK). To construct the targeting vector a recombineering system described in [33] was used. The resulting targeting vector contained exons 2–5 (out of 8 present in the gene), where exons 2–3 were flanked with loxP sites, and the neo cassette, flanked with FRT sites, was placed upstreams of the second loxP site. Targeting vector also contained MCI-TK cassette for positive selection of the transformed 129/Sv ES cells that were screened for genomic integration of the vector with Southern blotting and PCR. The positive clone was injected into C57BL/6J blastocysts to generate chimeric mice that were later intercrossed to generate *Aspn*^*fneo/+*^ heterozygous animals. They were then crossed with FLPeR mice (Jackson Laboratory, Bar Harbor, ME) to remove the neo cassette and give *Aspn*^*f/+*^ mice. Finally, they were crossed with *EIIa-Cre* transgenic mice (Jackson Laboratory, Bar Harbor, ME) to remove exons 2–3 of the targeted *Aspn* allele to create *Aspn*^*-/+*^ mice (Fig 1A). Primer sequences in Fig 1 are: P1 5’-ACTTCATTTTAACTTCCTTTACTGAGA-3’, P2 5’-AAACAGCTGGGTCTGTCCAT-3’, P3 5’-CCGGCTGCATGTTTATTTTC-3’.

### Skin histology and immunohistochemistry

Mouse skins were fixed in HistoChoice (Sigma) for 24 h, dehydrated, embedded in paraffin and sectioned. Sections were stained with Masson Trichrome (Sigma) or processed for immunohistochemistry: rehydrated sections were washed 2 x 5 min in Tris-buffered saline (TBS) plus 0.025% Triton X-100 and blocked with 10% goat serum and 1% bovine serum albumin (BSA) for 2 h. Sections were then incubated for 16 h at 4°C with rat anti-mouse CD31 (Optistain, clone SZ31) or rat anti-mouse F4/80 (BioRad, clone CI:A3-1) diluted to 10 μg/mL in 1% BSA in TBS. Sections were rinsed 2 x 5 min with TBS plus 0.025% Triton X-100 and quenched with 0.3% hydrogen peroxide in TBS for 15 min. After rinsing with TBS, sections were stained with ultrasensitive ABC staining kit (ThermoFisher). Sections were washed 3 x 5 min with TBS and developed using diaminobenzidine peroxidase substrate (Vector Labs).

### Immunoblots

Dorsal skin samples were boiled in SDS-PAGE loading buffer for 10 min. The proteins were run on 4–12% Bis-Tris reducing SDS-PAGE (ThermoFisher) and transferred to a nitrocellulose membrane. The membrane was blocked with 5% BSA in TBS, rinsed in wash buffer (TBS with 0.1% Tween-20) and immunoblotted in the incubation buffer (TBS with 0.1% Tween-20 and 1% BSA) using the following primary antibodies diluted to 1 μg/mL: anti-asporin (Abcam, ab31303), anti-decorin (kind gift from Professor Åke Oldberg, Lund University), anti-biglycan (LF106, kind gift from Professor Larry Fisher, NIH), anti-beta-actin (ab6276, Abcam). Membranes were washed 3 x 5 min and incubated with the corresponding secondary HRP-conjugated antibodies (all from GE Healthcare) diluted to 0.1 μg/mL for 1 h. Membranes were washed 3 x 5 min and developed with Luminata Forte (Millipore) using a CCD camera.

### Electron microscopy

Dorsal skin samples were shaved and processed for transmission electron microscopy as described [34]. Collagen fibril thickness was quantified using images from three reticular dermis regions per mouse, at 50,000x magnification, using Adobe Photoshop.

### Skin tensile strength determination

Mice were euthanized and kept on ice before shaving and gently removing the dorsal skin samples. Two parallel strips of skin (3 mm x 30 mm) were punched out in the craniocaudal direction, symmetrically on the left and right side of the spine. The samples were washed briefly in PBS, the central 5 mm were wrapped in PBS soaked gauze and the ends were allowed to dry for 90 min while keeping the central part moist with PBS. The dry ends were glued onto clamps using cyanoacrylate glue and after curing for 40 min the sample was placed in a PBS bath and mechanically tested (200-N tensile stage, petri dish version, Deben, Suffolk, UK). The test consisted of 4 preconditioning cycles to 15% strain, followed finally by a stretch to failure. All tests were at a rate of 6 mm/min with data recorded at 10 Hz. Sample width and length were measured by microscopy images. To avoid mechanically damaging the strips, thickness was measured with a constant-pressure caliper (model 293-334-30, Mitutoyo, Kawasaki, Japan) on a piece of skin adjacent to the tested strip. The failure test was analyzed for strain, stress and energy at both the yield point and at failure. The peak modulus was determined by a moving fit over a 10% strain range. Yield was defined as the first point where the modulus dropped below 50% of the peak value. For each animal, results for the two strips were averaged and groups were compared by unpaired two-tailed t-test with unequal variance.

### Collagen molecular phenotype (mass spectrometry)

Dorsal skin collagen was defatted with chloroform/methanol (3:1 v/v). Collagen was solubilized by 3% acetic acid extraction at 4°C for 24 hrs or heat denaturation in SDS-PAGE sample buffer. The extracts were run on 6% SDS-PAGE and stained with Coomassie Blue. Bands corresponding to collagen α1(I) and α2(I) chains were cut out and processed for mass spectrometry using trypsin in-gel digestion, without reduction or alkylation. Collagen peptides were analyzed with LC-MS using an LTQ XL ion trap mass spectrometer (ThermoFisher) equipped with in-line liquid chromatography using a C4 5um capillary column (300um x 150mm; Higgins Analytical RS-15M3-W045) eluted at 4.5ul min. The LC mobile phase consisted of buffer A (0.1% formic acid in MilliQ water) and buffer B (0.1% formic acid in 3:1 acetonitrile:n-propanol v/v). The LC sample stream was introduced into the mass spectrometer by electrospray ionization (ESI) with a spray voltage of 4kV. Proteome Discoverer search software (Thermo Scientific) was used for peptide identification using the NCBI protein database. Proline and lysine modifications were examined manually by scrolling or averaging the full scan over several minutes so that all of the post-translational variations of a given peptide appeared together in the full scan.

### Hydroxyproline determination

Dorsal skin samples were weighed and hydrolyzed in 6 M HCl for 4 hours at 120°C at 2 atm. Hydroxyproline was quantified as described earlier [35] and normalized to total tissue weight.

### Reverse transcriptase- and real-time qPCR

Total RNA was isolated from skin mice using TRIzol reagent (ThermoFisher). 500 ng RNA was used for reverse transcription reaction using Superscript VILO (ThermoFisher). Real-time qPCR was conducted using Taqman probes and Taqman Gene Expression Master Mix (ThermoFisher) in an Applied Biosystems 7300 qPCR equipment. Gene expression was normalized to *Actb* transcripts. The Taqman probe list is presented in S1 Table.

### Glycosaminoglycan analysis

Skins were lyophilized, weighed, and processed for GAG analyses as described in [34]. The proteins were degraded for 16 h at 55°C with 200 μg/ml pronase in 50 mm Tris/HCl, pH 8, 1 mM CaCl_2_, and 1% Triton X-100. After heat inactivation of the protease, MgCl_2_ was added to 2 mM and benzonase was added to a final 20 sigma units/ml and incubated for 2 h at 37°C. DNAase was heat-inactivated, NaCl was added to a final 0.1 M, and GAGs were purified by batch incubation with a DE52 anion exchange gel, followed by the transfer of the gel to disposable spin centrifuge tubes (Costar 8163). The gel was washed with buffer 1 (50 mM Tris/HCl, pH 8, 0.1 M NaCl, and 0.1% Triton X-100), buffer 2 (50 mM NaAc, pH 4, 0.1 M NaCl, and 0.1% Triton X-100), water, and 0.1 M NH_4_HCO_3_. Elution was achieved by 2 M NH_4_HCO_3_. After lyophilization, GAGs were provisionally and roughly estimated by carbazole [36]. Then, 500 ng GAGs were subjected to chondroitinase ABC (Sigma) overnight at 37°C in 20 μl 50 mM NH_4_OAc and 0.1 mg/ml BSA, containing 10 mIU enzyme, or chondroitinase B (R&D Systems) overnight at 37°C in 20 μl 50 mM Tris pH 7.5, 50 mM NaCl, 4 mM CaCl_2_, and 0.1 mg/ml BSA, containing 5 mIU enzyme, or with a mixture of heparinases (in-house preparation, purified from *E*.*coli*, stably singularly transfected with the pET-15b vector containing heparinase I, or vector pET-19b containing heparinase II or III, as provided by Professor Jian Liu (University of North Carolina; incubation overnight at 37°C in 20 μl 40 mM NaAc pH 7.0, 2 mM CaAc, 0.1 mg/ml BSA, containing 2 mIU of each enzyme). The samples were boiled and the supernatant was dried. Fluorophore-labelling of the resulting disaccharides was performed by adding 10 μl of 20 mM re-purified 2-aminoacridone (AMAC, Sigma), followed by a 20 min incubation at room temperature before the addition of 10 μl of 1 M NaBH_3_CN and incubation at 45°C for 16 h [37]. Pre-column AMAC-labeled disaccharides were analyzed with HPLC-fluorescence as described previously, with slight modifications [37]. Briefly, 20 μl samples were diluted to 100 μl in running buffer (98% A: NH_4_OAc, 60 mM, pH 5.6, and 2% B: MeCN) and injected (2 μl) onto an XBridge BEH Shield RP18 (2.1x100 mm, 2.5 μm). Disaccharides were separated using a 39 min gradient run at 0.35 ml/min (0–1 min: 98% A, 1–3 min: 98–96% A, 3–26 min: 96–85% A, 26–28 min: 85–10% A, 28–32 min: 10% A, 32–34 min: 10–98% A, 34–39 min: 98% A) on a Thermo Scientific UltiMate 3000 Quaternary Analytical system with an FLD-3400RS fluorescence detector (excitation λ = 428 and emission λ = 525). The column was kept at 30°C to improve performance and reproducibility. Quantification was done by comparison to known weight of standard disaccharides (Iduron, UK), mock-treated in the same buffers and enzymes as the samples in each series of runs.

### Semi-quantitative analysis of decorin and biglycan protein cores

Proteins were extracted from two-month old mice skin (n = 4 per genotype) in a dissociating buffer (4 M guanidine chloride, 50 mM NaAc pH 5.5, 0.1% Triton X-100, complete protease inhibitor cocktail (Roche)) by consecutive use of a Polytron- and Potter-type homogenizers. After overnight incubation the extracts were spun down and the supernatant exchanged against a denaturating buffer (8 M urea, 50 mM NaAc pH 5.5, 0,2 M NaCl, 0.1% Triton X-100). Proteins were quantified by Bradford method (BioRad) and equal amount of proteins were used in anion-exchange purification of proteoglycans on DE-52 (Whatman), eluted by of 2M ammonium bicarbonate, 0.1% Triton X-100. The recovery from the DE-52 purification was assessed by co-incubation of ^35^S-labeled GAGs, which were protease-treated, with the skin extracts. The recovery of the labeled internal control was very similar among the eight samples (coefficient of variation = 11%). SDS-PAGE electrophoresis was done using stain-free gels (BioRad) and total proteins were visualized directly using the Chemi Doc Touch Imaging System (BioRad). For immunoblotting the samples were digested with chondroitinase ABC (as described in Glycosaminoglycan Analysis). Immunoblots were performed as described above.

## Results

### Generation of *Aspn*^*-/-*^ mice

Our gene-targeting construct was designed to remove exons 2–3 in the *Aspn* gene where exon 2 contains the translation start site and 23 bp of upstream promoter sequence. First, we flanked exons 2–3 with two direct repeats of loxP sites, where the second loxP site was preceded by a neo cassette flanked with FRT sites. The gene-targeted mice were then bred with FLPeR mice in order to remove the neo cassette, and then with *EIIa-Cre* mice to remove exons 2–3 (Fig 1A). The resulting *Aspn*^*+/-*^ mice were backcrossed with C57BL/6 mice for ten generations. When intercrossed, *Aspn*^*+/-*^ mice generated *Aspn*^*+/+*^, *Aspn*^*+/-*^ *and Aspn*^*-/-*^ pups at normal Mendelian ratio. Excision of exons 2 and 3 was verified by genotyping (Fig 1B). No full size or fragments of asporin protein were present in *Aspn*^*-/-*^ mice, confirming the functional ablation of the asporin gene (Fig 1C). All mice had normal fertility and external appearance. Five *Aspn*^*-/-*^ mice were kept until 15 months of age and showed normal behavior. Histological and necroscopical analyses of 2 month-old *Aspn*^*-/-*^ mice showed no gross abnormities in aorta, esophagus, intestine, kidney, liver, skeletal muscle, spleen and trachea.

### Skin histology and electron microscopy

**Fig 2:**
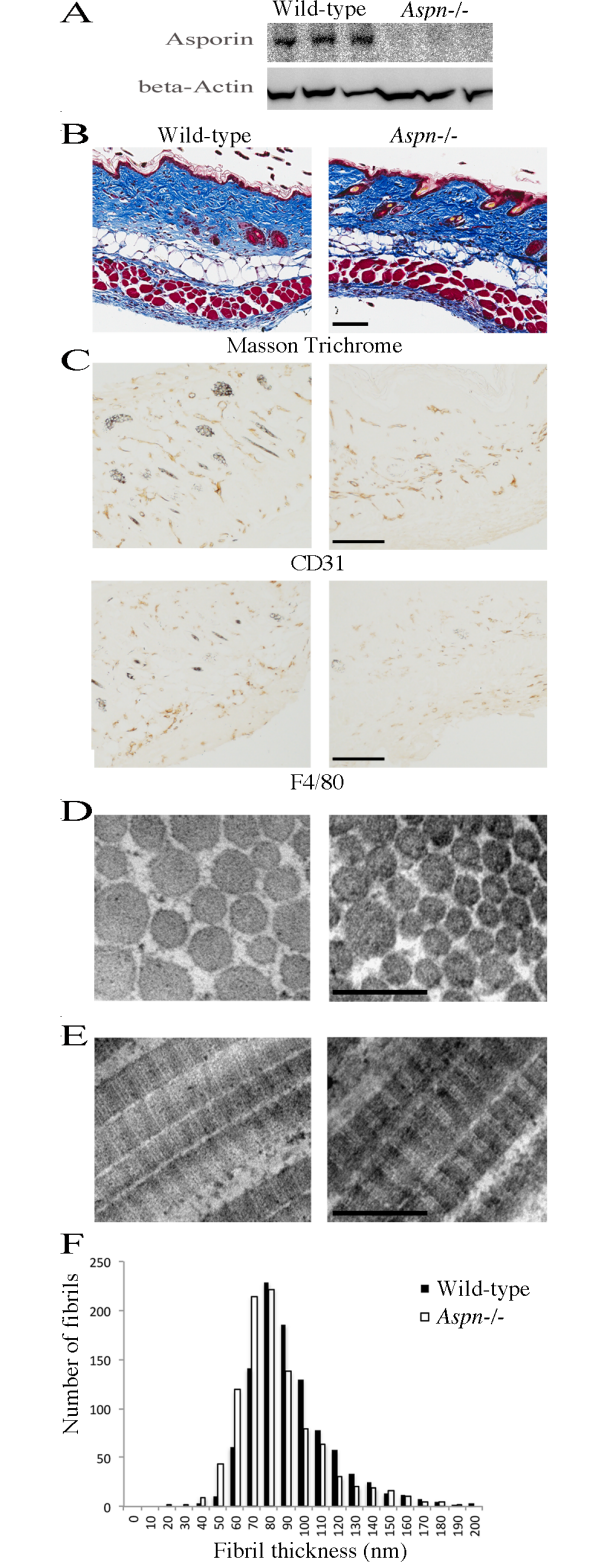
Skin phenotype of *Aspn*^-/-^ mice. (A) Asporin immunoblotting of dorsal skin extracts from three wild-type and three *Aspn*^*-/-*^ mice. (B) 2 month-old female dorsal skins were analyzed histologically by staining with Massson Trichrome, or (C) by immunohistochemistry by staining for the presence of CD31-positive blood vessels and F4/80-positive macrophages; bars in A and *B* are 100 μm. Similar results were obtained from analyses of six mice from each genotype. (D) Transmission electron microscopy on cross-sectioned collagen fibrils in reticular dermis. (E) Transmission electron microscopy on longitudinally oriented collagen fibrils in reticular dermis. Bars in *C* and *D* are 200 nm. (F) Quantification of collagen fibril diameter in reticular dermis. 1,000 fibrils were measured from electron microscopy images collected from six wild-type and six *Aspn*^-/-^ mice.

Asporin protein expression in dorsal skin was confirmed by immunoblotting SDS extracts. Also, absence of asporin protein in *Aspn*^*-/-*^ mice skin was confirmed (Fig 2A). In 2 month-old female *Aspn*^*-/-*^ mice Masson Trichrome staining did not reveal any apparent abnormities in the structure of dermis or hypodermis (n = 6 per genotype) (Fig 2B), and there were no apparent differences in the density of blood vessels (CD31-positive cells) or macrophages (F4/80-positive cells) (Fig 2C). We did not observe any histological abnormities in male mice skin either (n = 3 per genotype, S1 File). Observed in transmission electron microscopy, *Aspn*^*-/-*^ reticular dermis had regularly contoured collagen fibrils (Fig 2D) with typical collagen banding pattern (Fig 2E). Collagen fibril diameter was 7% thinner in *Aspn*^*-/-*^ skin (Fig 2F) with the average diameter of 88 ± 5 nm in wild-type (n = 6) and 82 ± 10 nm in *Aspn*^*-/-*^ skin (n = 6; p = 0.23, Welch unpaired two-tailed t-test). Assessed by hydroxyproline determination, collagen quantity per wet tissue weight was not significantly changed (wild-type 67 ± 13 μg/mg, n = 5; *Aspn*^*-/-*^ 58 ± 5 μg/mg, n = 5; p = 0.15, Welch unpaired two-tailed t-test).

### Skin mechanical testing

Next, the general mechanical properties of *Aspn*^*-/-*^ skin were assessed. In four-month old female *Aspn*^*-/-*^ mice the skin toughness, or energy required to achieve failure, was almost 75% greater than in wild-type mice (Table 1). Other mechanical parameters, including failure stress and strain, were not significantly different but also tended toward superior strength of the *Aspn*^*-/-*^ skin. Note that the parameters at yield are almost identical in both genotypes, indicating that most of the difference occurred after the yield point.

**Table 1:** Skin mechanical testing.

	Wild-type		*Aspn*^*-/-*^		
	avg	sd	avg	sd	p value
Sample Thickness (mm)	0.48	0.11	0.39	0.03	0.13
Modulus (MPa)	13.8	7.3	16.1	6.4	0.59
Yield Strain (%)	47	7.2	46	9.2	0.88
Yield Stress (MPa)	4.0	1.8	4.4	1.2	0.67
Yield Energy (MJ/m^3^)	0.75	0.32	0.85	0.39	0.63
Failure Strain (%)	50	7	62	15	0.11
Failure Stress (MPa)	4.2	1.9	5.3	0.8	0.22
Failure Energy (MJ/m^3^)	0.89	0.38	1.55	0.46	0.022

Uniaxial tensile material properties in the craniocaudal direction, from duplicate measures on four-month old wild-type and *Aspn*^*-/-*^ dorsal skin samples (n = 6 per genotype; avg = average, sd = standard deviation).

### Skin collagen molecular phenotype

Because asporin is a collagen-binding protein we investigated if asporin deficiency could result in a changed molecular phenotype of collagen. Two-month old skin collagen was extracted with SDS or acetic acid and the collagen band pattern on Coomassie-stained SDS-PAGE was analyzed. We did not observe any differences between *Aspn*^*-/-*^ and wild-type collagen (S1 Fig). We also used mass spectrometry to assess lysyl oxidase-mediated and lysyl hydroxylase-mediated modifications of collagen lysines involved in cross-linking. We could not detect any differences in the oxidation status of α1(I) chain C-telopeptidal lysine, and no differences in the hydroxylation status of α1(I) helical Lys-87, or in α2(I) helical Lys-90 (S1 Fig).

### Skin gene expression

To assess the general extracellular matrix gene expression qPCR on selected gene transcripts was performed (Table 2). *Aspn*^*-/-*^ skin had an increased expression of collagens (*Col1a1*, *Col1a2*, *Col3a1*), lysyl oxidases (*Lox*, *Loxl2*), collagenases (*Mmp2*, *Mmp3*), the collagen secretion-related *Serpinh1* and *Mia3*, and the collagen fibril-modulating *Lum*. The expression of *Plod2*, the collagen telopeptide-targeted lysine hydroxylase, was decreased. The expression of *Fmod* and *Fbn1* was unaltered.

**Table 2:** Quantification of gene transcripts using qPCR.

	Relative transcript amount in *Aspn*^-/-^ (fold of wild-type)		Wild-type		*Aspn*^*-/-*^	
		p value	dCt	sd dCt	dCt	sd dCt
*Col1a1*	6.86	0.00004	-0.21	0.71	-2.99	0.27
*Col1a2*	3.96	0.00004	2.63	0.28	0.64	0.46
*Col3a1*	3.52	0.00008	1.85	0.37	0.03	0.41
*Fmod*	1.57	0.3	6.82	1.03	6.17	0.87
*Lox*	3.52	0.0002	5.73	0.34	3.91	0.54
*Loxl2*	1.41	0.07	5.28	0.45	4.78	0.28
*Plod2*	0.23	0.004	5.32	0.18	7.43	1.19
*Lum*	2.21	0.03	7.68	0.87	6.54	0.47
*Fbn1*	1.02	0.9	3.91	0.57	3.88	0.16
*Mmp2*	2.41	0.002	4.15	0.47	2.88	0.38
*Mmp3*	3.19	0.0005	7.87	0.50	6.19	0.45
*Serpinh1*	2.68	0.0001	5.36	0.31	3.94	0.35
*Mia3*	1.91	0.001	8.81	0.31	7.88	0.31

Dorsal skin RNA from two-month old female mice (n = 5 per genotype) was used to determine mRNA levels of selected extracellular matrix-related genes. *Aspn*^-/-^ mRNA levels are presented as percentage of wild-type mRNA levels, all normalized to *Actb*. P values were calculated using Student’s unpaired two-tailed t-test. Also shown are delta Ct (dCt) and standard deviation (sd dCt).

### Skin glycosaminoglycan analysis

**Fig 3:**
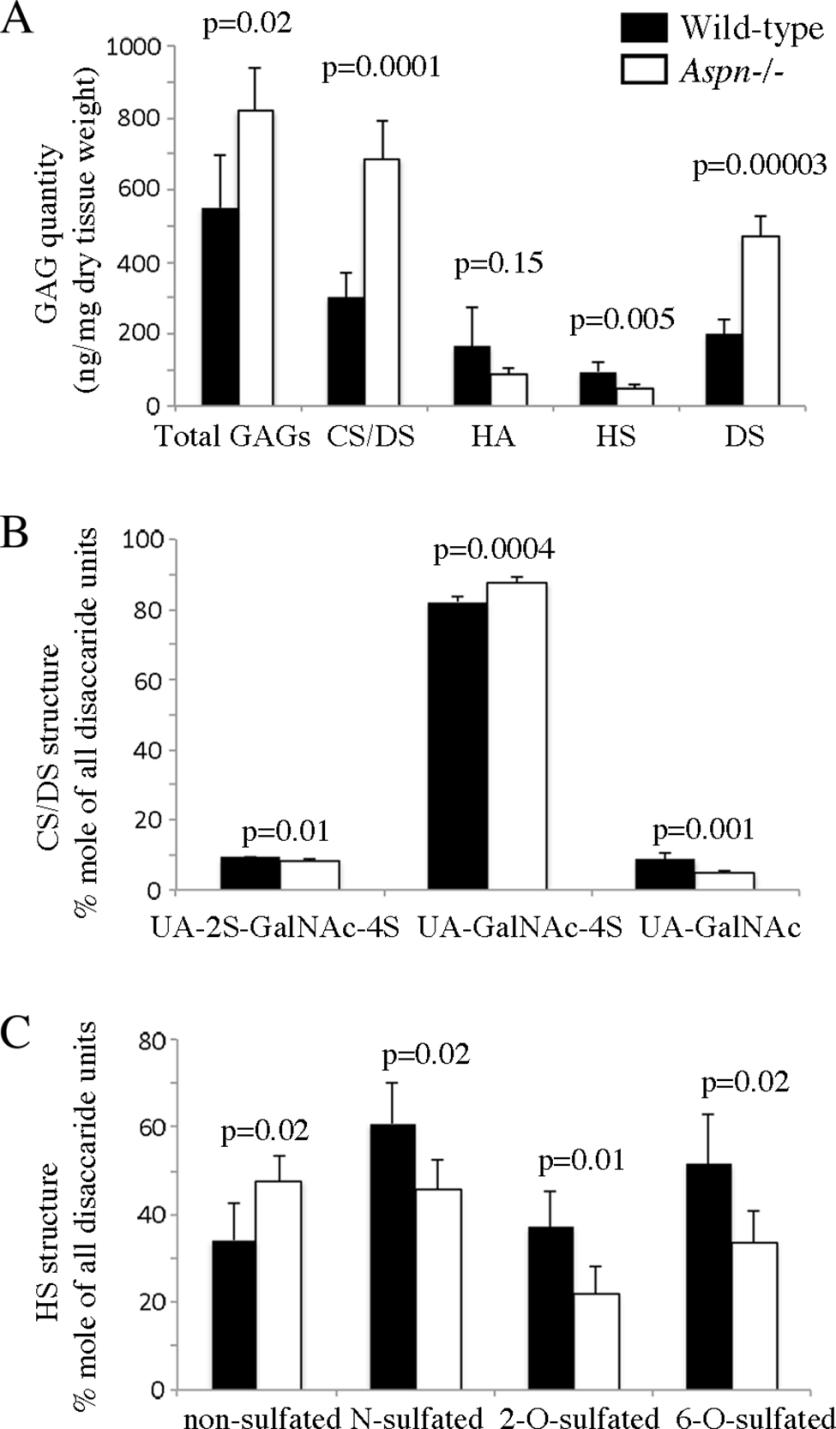
Skin glycosaminoglycan (GAG) analysis. GAGs were purified from two-month old dorsal skin (n = 5 per genotype) and digested with chondroitinase ABC or chondroitinase B or a mixture of heparinases. The resulting disaccharides were fluorescently labeled, separated by HPLC and quantified. (A) Total GAGs are the sum of chondroitin/dermatan sulfate (CS/DS), hyaluronic acid (HA), and heparan sulfate (HS). (B) Compositional analysis of CS/DS chains. UA = unsaturated hexuronic acid obtained after chondroitinases digestion, either unsulfated or 2-*O*-sulfated (UA-2S); GalNAc = *N*-acetyl-galactosamine either unsulfated or 4-*O*-sulfated (GalNAc-4S). (C) Compositional analysis of HS chains. The measured HS disaccharides were grouped according to the sulfation position: non-sulfated = UA-GlcNAc; *N*-sulfated = UA-GlcNS + UA-2S-GlcNS + UA-GlcNS-6S + UA-2S-GlcNS-6S; 2-*O*-sulfated = UA-2S-GlcNAc + UA-2S-GlcNAc-6S + UA-2S-GlcNS + UA-2S-GlcNS-6S; 6-*O*-sulfated = UA-GlcNAc-6S + UA-2S-GlcNAc-6S + UA-GlcNS-6S + UA-2S-GlcNS-6S. UA = unsaturated hexuronic acid obtained after heparinases digestion, either unsulfated or 2-*O*-sulfated (UA-2S); Glc = glucosamine either *N*-acetylated (GlcNAc) or *N*-sulfated (GlcNS) which can be both O-unsulfated or 6-*O*-sulfated (GlcNAc-6S and GlcNS-6S). Error bars show standard deviation.

To characterize GAG content and structure in two-month old wild-type (n = 5) and *Aspn*^*-/-*^ skin (n = 5), we utilized HPLC quantification and identification of disaccharides obtained after chondroitinase ABC- or B- or heparinases-digestion (representative chromatograms are presented in S2 Fig). *Aspn*^*-/-*^ mice had higher chondroitin/dermatan sulfate (CS/DS) (wild-type 302 ± 65 ng/mg dry tissue; *Aspn*^*-/-*^ 685 ± 105 ng/mg) but lower heparan sulfate (HS) content (wild-type 96 ± 25 ng/mg; *Aspn*^*-/-*^ 49 ± 12 ng/mg). Hyaluronic acid quantification showed no differences (Fig 3A). Compositional analysis of CS/DS chains revealed a decrease in non-sulfated disaccharide structures UA-GalNAc (wild-type 8.7 ± 1.7%; *Aspn*^*-/-*^ 4.7 ± 0.5%) compensated by an increase in the 4-*O*-sulfated disaccharide structures UA-GalNAc-4S (wild-type 82.2 ± 1.6%; *Aspn*^*-/-*^ 87.7 ± 1.4%). The 2- and 4- disulfated structures UA-2S-GalNAc-4S were slightly reduced from 9.1 ± 0.4% in wild-type to 8.2 ± 0.5% in *Aspn*^*-/-*^ (Fig 3B). In order to measure the content of iduronic acid in CS/DS we performed digestions with chondroitinase B, which cleaves only N-acetyl galactosamine-iduronic acid linkages [38]. The analyzed disaccharides must come from at least two adjacent iduronic acid-containing repeats in the native chain, most likely reflecting the long iduronic acid blocks present in the skin decorin and biglycan CS/DS chain. These iduronic acid-containing structures were increased in *Aspn*^*-/-*^ skin (wild-type 200 ± 40 ng/mg dry tissue; *Aspn*^*-/-*^ 470 ± 59 ng/mg; Fig 3A), showing that they constitute the majority of CS/DS chains in both genotypes (66% in wild-type and 69% in *Aspn*^*-*/-^), in agreement with a previous report [39]. As expected, these iduronic acid blocks contain mostly 4-sulfated iduronic-N-acetyl galactosamine (87 and 88% in wild-type and in *Aspn*^*-/-*^, respectively) and the remaining moieties are 2- and 4- disulfated.HS chains in *Aspn*^-/-^ skin were less sulfated in all three positions, i.e. glucosamine N-sulfated and 6-*O*-sulfated, and hexuronic 2-*O-*sulfated (Fig 3C). In summary, *Aspn*^*-/-*^ skin contained more CS/DS and less HS, and HS was less sulfated.

### Semi-quantitative assessment of decorin and biglycan

**Fig 4:**
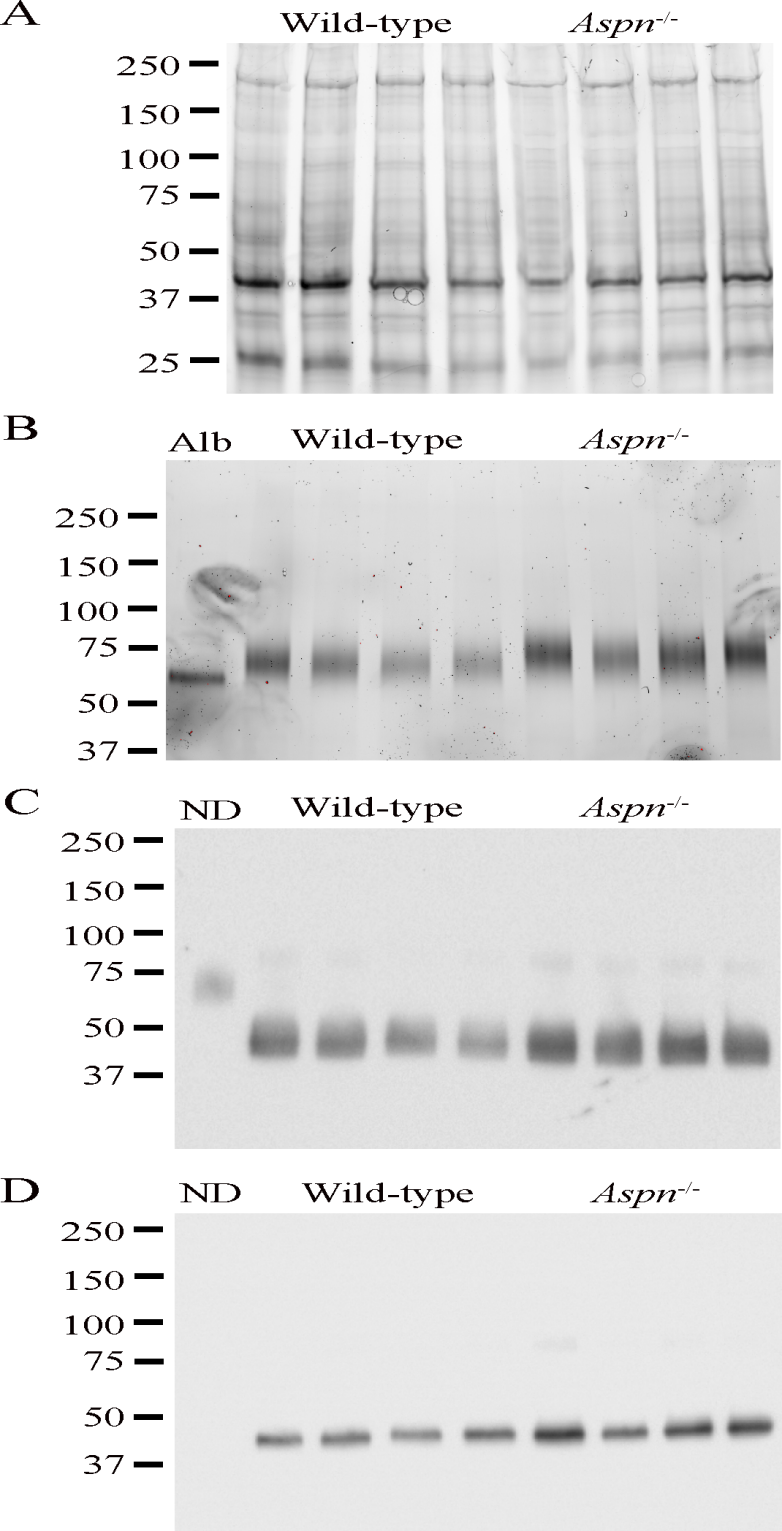
Semi-quantitative assessment of decorin and biglycan. Dorsal skin proteins were extracted with guanidine- and dialyzed against urea-containing buffers. Total protein quantity was assessed by Bradford, and by gel imaging (A). Equal protein quantities were applied on anion-exchange beads and proteoglycans were eluted and imaged on gel (B); *Alb* is albumin control. Next, proteoglycan samples were digested with chondroitinase ABC and immunoblotted for decorin (C) and biglycan (D) to assess the quantities of the respective protein cores; *ND* is non-chondroitinase ABC-digested sample control (not visible in biglycan samples probably due to low quantity and smeary distribution).

To assess the quantities of skin proteoglycans we extracted proteins from dorsal skin samples (n = 4 per genotype) and applied similar total protein quantities (Fig 4A) to anion-exchange. The eluted total proteoglycans (Fig 4B) appeared twofold more abundant in *Aspn*^*-/-*^ skin. To verify our findings and to identify the different proteoglycans we digested the samples with chondroitinase ABC and immunoblotted for decorin and biglycan. *Aspn*^*-/-*^ skin contained approximately twofold more decorin (Fig 4C) and twofold more biglycan (Fig 4D).

## Discussion

Collagens can be arranged and cross-linked in different ways, which is apparent from the variety of collagen fibril phenotypes in different tissues and organs. The question of mechanisms behind these processes remains intriguing, but several seemingly tissue-specific modulators of collagen fibrillogenesis have been identified *in vitro* and *in vivo*, especially among the SLRP family of proteins [1, 2]. In order to examine the role of the less investigated SLRPs we have characterized the *in vivo* extracellular matrix phenotype in a novel, SLRP-deficient, *Aspn*^*-/-*^ mouse. In earlier studies asporin gene polymorphism has been associated with human knee osteoarthritis and lumbar disc degeneration [20, 40]; we were therefore intrigued that *Aspn*^*-/-*^ mice did not show any apparent skeletal abnormities, even at histological examination. We suggest that the polymorphisms described in the previous papers [20, 40] have a different effect than total gene deletion. More studies are needed to elucidate the phenotypic impact of the different *ASPN* gene mutations.

In this paper we focused on skin phenotype–this is mainly because we, in this study, and other groups earlier [21, 22], detected asporin in dermis, and because we observed no abnormities in other major organs in *Aspn*^*-/-*^ mice. In addition, we observed a changed biomechanical phenotype in *Aspn*^*-/-*^ skin, which prompted the skin ECM analyses conducted in this study.

The skin extracellular matrix in *Aspn*^*-/-*^ and wild-type mice was comparable in histological examinations, i.e. the relative amount of dermis and hypodermis, and the amount of blood vessels appeared similar in both genotypes. We observed no signs of inflammation, judging from the unaltered macrophage amounts.

Since asporin interacts with collagen and inhibits its fibrillogenesis *in vitro* [17] we conducted a detailed investigation of the molecular collagen phenotype in *Aspn*^*-/-*^ skin. Collagen fibrils examined in an electron microscope were 7% thinner in *Aspn*^*-/-*^ skin; this change, however, was not statistically significant and the fibrils were morphologically regular, in contrast to the fibril phenotypes reported in other SLRP-deficient mice, e.g. *Dcn*^*-/-*^ and *Lum*^*-/-*^ [6, 9]. Furthermore, the collagen band pattern on Coomassie-stained SDS-PAGE appeared normal, suggesting no defects in collagen post-translational modifications, e.g. proline hydroxylation. Despite a decrease in the amount of lysyl oxidase, the collagen α1(I)- and α2(I)-chain lysines involved in cross-linking were not modified differently than in wild-type collagen, in contrast to the phenotype reported in *Fmod*^*-/-*^ mice [3]. Summarizing the collagen phenotype studies, we could detect only a small, statistically non-significant, difference in the fibril diameter in the *Aspn*^*-/-*^ skin collagen. It is possible that the homologue decorin, which we found approximately doubled in *Aspn*^*-/-*^ skin, binding to the same site on collagen as asporin [17] can compensate for the asporin deficiency, thus ensuring an essentially normal collagen fibril phenotype.

Despite the almost normal *Aspn*^*-/-*^ skin collagen fibril phenotype we observed a robust transcript increase of collagens (*Col1a1*, *Col1a2*, *Col3a1)*, collagen secretion-related *Serpinh1* and *Mia3*, collagen cross-linking-related lysyl oxidases (*Lox*, *Loxl2*), the collagen fibrillogenesis-modulating SLRP *Lum*, and the collagenases *Mmp2 and Mmp3*. Altogether, the expression data could be explained by an overall augmented ECM turnover, whose effect, however, on the overall ECM structure observed by histology and electron microscopy appears minute.

While we did not detect any major changes in the overall skin and ECM structure or in collagen molecular phenotype we observed a major change in GAG content and structure. In the ECM a major component are chondroitin/dermatan (CS/DS) proteoglycans versican, decorin and biglycan. In *Aspn*^*-/-*^ skin, CS/DS was more than doubled and the overall CS/DS sulfation was increased. The doubling of CS/DS corresponds well with the increased amount of decorin and biglycan protein cores in *Aspn*^*-/-*^ skin. This could reflect a compensatory mechanism triggered by deficiency of one SLRP, as was the case in e.g. fibromodulin knockout mice [7]. Interestingly, HS amount decreased in *Aspn*^*-/-*^ skin, and was considerably less sulfated, which could impact the cellular response to different growth factors. Overall, these changes in GAG content and structure could reflect a general cellular response to a changed extracellular matrix microenvironment. Our attempts at finding the mechanisms behind this response have so far not been fruitful.

The above-described changes in the ECM phenotype translate into a mechanically tougher skin in *Aspn*^*-/-*^ mice. The strain-stress curves obtained from biomechanical analyses suggest that the difference between *Aspn*^*-/-*^ and wild-type skin did not manifest in the initial elastic region where the deformation is reversible, but rather in the plastic region between the yield and the failure point, where sliding and possibly rupture of collagen fibrils lead to irreversible damage. We suggest that a compound effect of slightly thinner collagen fibrils and the large difference in GAG composition could both contribute to this biomechanical phenotype. While perhaps counterintuitive, the correlation of thinner collagen fibrils and higher mechanical strength has been described before, e.g. where decorin inhibited fibril assembly but increased fibril strength [41], and where thinner fibrils in 3D gels conferred increased tensile strength [42], possibly through promoting more interconnected fibrillar network (increased surface-to-volume ratio) [43]. Regarding GAGs’ influence on the mechanical properties, several papers argue against that possibility in a number of *in vitro* or *ex vivo* studies [41, 44, 45]. These studies, however, were performed on ligaments, where collagen fibers are largely aligned in parallel and have therefore limited compressive force components. Skin, unlike ligaments, will likely have more compression between fibers even under tensile load. This compression is likely to be influenced by the water-retaining GAGs, and in particular CS/DS. We suggest that the changed GAG composition and structure, as well as the increased amounts of decorin and biglycan, in *Aspn*^*-/-*^ skin provide an explanation for the altered biomechanical properties.

## Supporting information

S1 Fig Collagen molecular phenotype. (A) Two-month old dorsal skin collagen was extracted with either SDS-PAGE loading buffer or 3% acetic acid, run on 6% SDS-PAGE and stained with Coomassie Blue. The major collagen bands are denoted on the right. Similar results were obtained by analyzing nine mice of each genotype. (B) Mass spectrometry analysis of modifications of prolines and lysines around collagen cross-linking sites. The analyzed peptides’ sequences are shown at the top of each panel. Mass spectra show similar oxidation status of both prolines and lysines in both wild-type (WT) and Aspn-/- (KO) mice.
(TIF)

S2 Fig GAG analysis chromatograms. HPLC disaccharide chromatograms of CS/DS (A), DS (B), HS (C). GAGs were purified and digested with chondroitinase ABC (A), chondroitinase B (B), or heparinases I+II+III (C), then AMAC-labeled and HPLC-separated. In (A) and (B) the disaccharide identity is: peak 1: UA-2S-GalNAc-4S (B structure); peak 2: UA-GalNAc-4S-6S (E structure); peak 3: UA-2S-GalNAc-6S (D structure); peak 4: UA-GalNAc-4S (A structure); peak 5: UA-GalNAc-6S (C structure); peak 6: HA-derived UA-GlcNAc; peak 7: UA-GalNAc (0 structure). In (C) the disaccharide identity is: peak 1: UA-2S-GlcNS-6S; peak 2: UA-GlcNS-6S; peak 3: UA-2S-GlcNS; peak 4: UA-2S-GlcNAc; peak 5: UA-GlcNS; peak 6: UA-2S-GlcNAc-6S; peak 7: UA-GlcNAc-6S; peak 8: UA-GlcNAc. Asterisks indicate unidentified peaks. Similar chromatograms were obtained with other samples from the respective genotype.
(TIF)

S1 Table List of Taqman probes used for qPCR skin gene expression analysis. (DOCX)

S1 File Masson Trichrome stainings of skin. Images from dorsal skin Masson Trichrome stainings. Three male and three female mice per genotype were used. ASPN WT are wild-type, ASPN KO are *Aspn*^*-/-*^.
(ZIP)

S2 File Raw data on skin biomechanics measurements. The data were collected as described in Materials and Methods, Skin tensile strength determination.
(XLSX)

S3 File Raw data on skin collagen fibril diameter measurements. The data were collected as described in Materials and Methods, Electron microscopy.
(XLSX)

S4 File Raw data from skin gene expression analysis by qPCR. The data were collected as described in Materials and Methods, Reverse transcriptase- and real-time qPCR.
(XLSX)
