# Supplementary figures and images for: Asporin-deficient mice have tougher skin and altered skin glycosaminoglycan content and structure
Source: PLoS One. 2017 Aug 31;12(8):e0184028. doi: 10.1371/journal.pone.0184028 (PMC5578652; doi:10.1371/journal.pone.0184028)

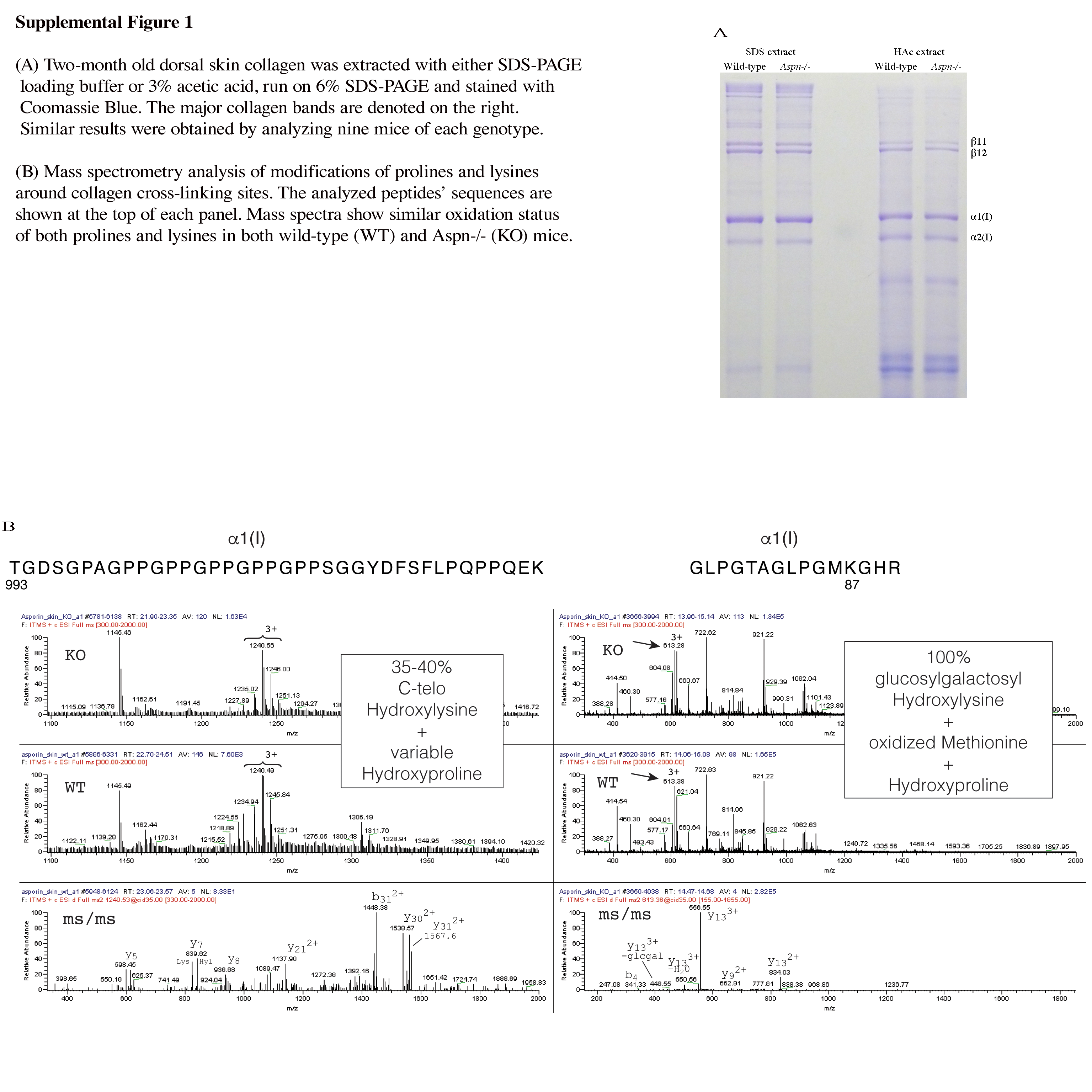

Supplement: S1 Fig — (A) Two-month old dorsal skin collagen was extracted with either SDS-PAGE loading buffer or 3% acetic acid, run on 6% SDS-PAGE and stained with Coomassie Blue. The major collagen bands are denoted on the right. Similar results were obtained by analyzing nine mice of each genotype. (B) Mass spectrometry analysis of modifications of prolines and lysines around collagen cross-linking sites. The analyzed peptides’ sequences are shown at the top of each panel. Mass spectra show similar oxidation status of both prolines and lysines in both wild-type (WT) and Aspn-/- (KO) mice. (TIF) [file pone.0184028.s001.tif]

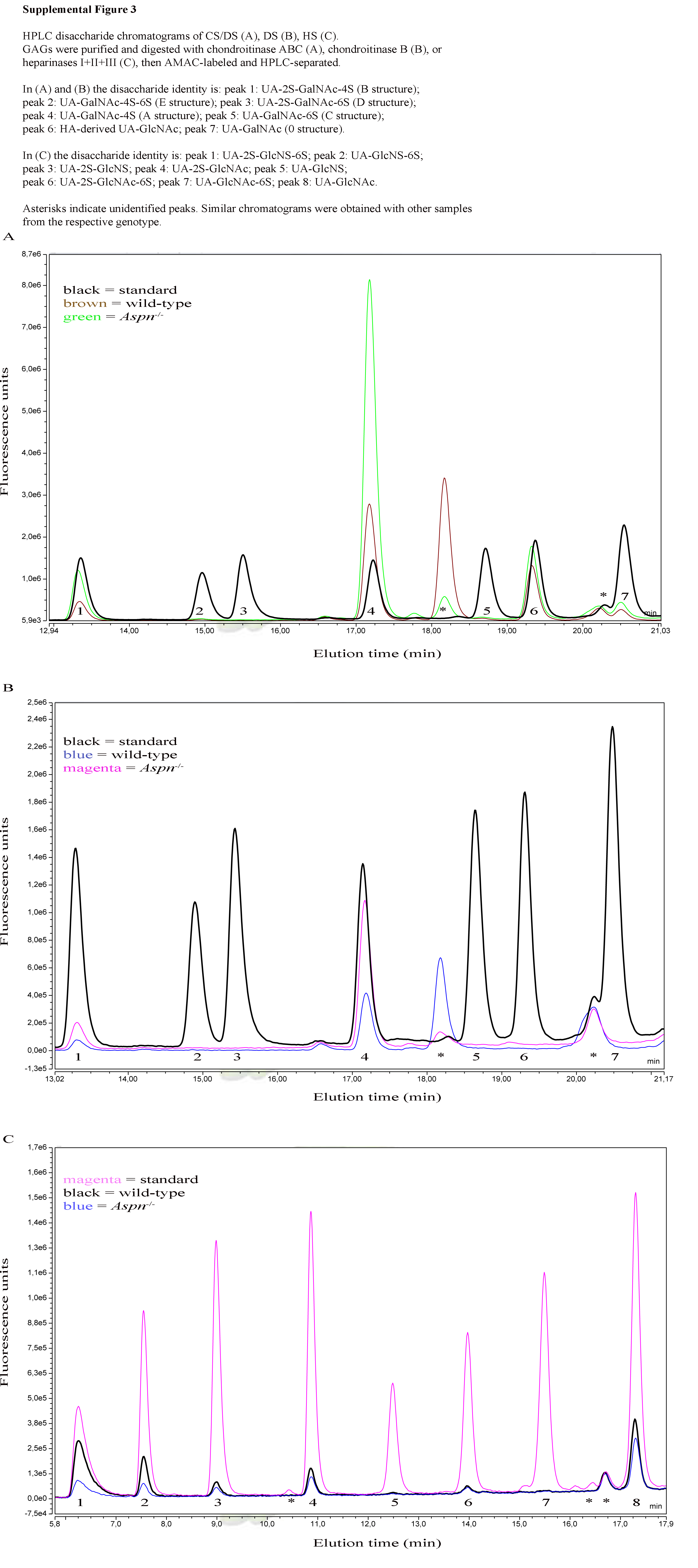

Supplement: S2 Fig — HPLC disaccharide chromatograms of CS/DS (A), DS (B), HS (C). GAGs were purified and digested with chondroitinase ABC (A), chondroitinase B (B), or heparinases I+II+III (C), then AMAC-labeled and HPLC-separated. In (A) and (B) the disaccharide identity is: peak 1: UA-2S-GalNAc-4S (B structure); peak 2: UA-GalNAc-4S-6S (E structure); peak 3: UA-2S-GalNAc-6S (D structure); peak 4: UA-GalNAc-4S (A structure); peak 5: UA-GalNAc-6S (C structure); peak 6: HA-derived UA-GlcNAc; peak 7: UA-GalNAc (0 structure). In (C) the disaccharide identity is: peak 1: UA-2S-GlcNS-6S; peak 2: UA-GlcNS-6S; peak 3: UA-2S-GlcNS; peak 4: UA-2S-GlcNAc; peak 5: UA-GlcNS; peak 6: UA-2S-GlcNAc-6S; peak 7: UA-GlcNAc-6S; peak 8: UA-GlcNAc. Asterisks indicate unidentified peaks. Similar chromatograms were obtained with other samples from the respective genotype. (TIF) [file pone.0184028.s002.tif]

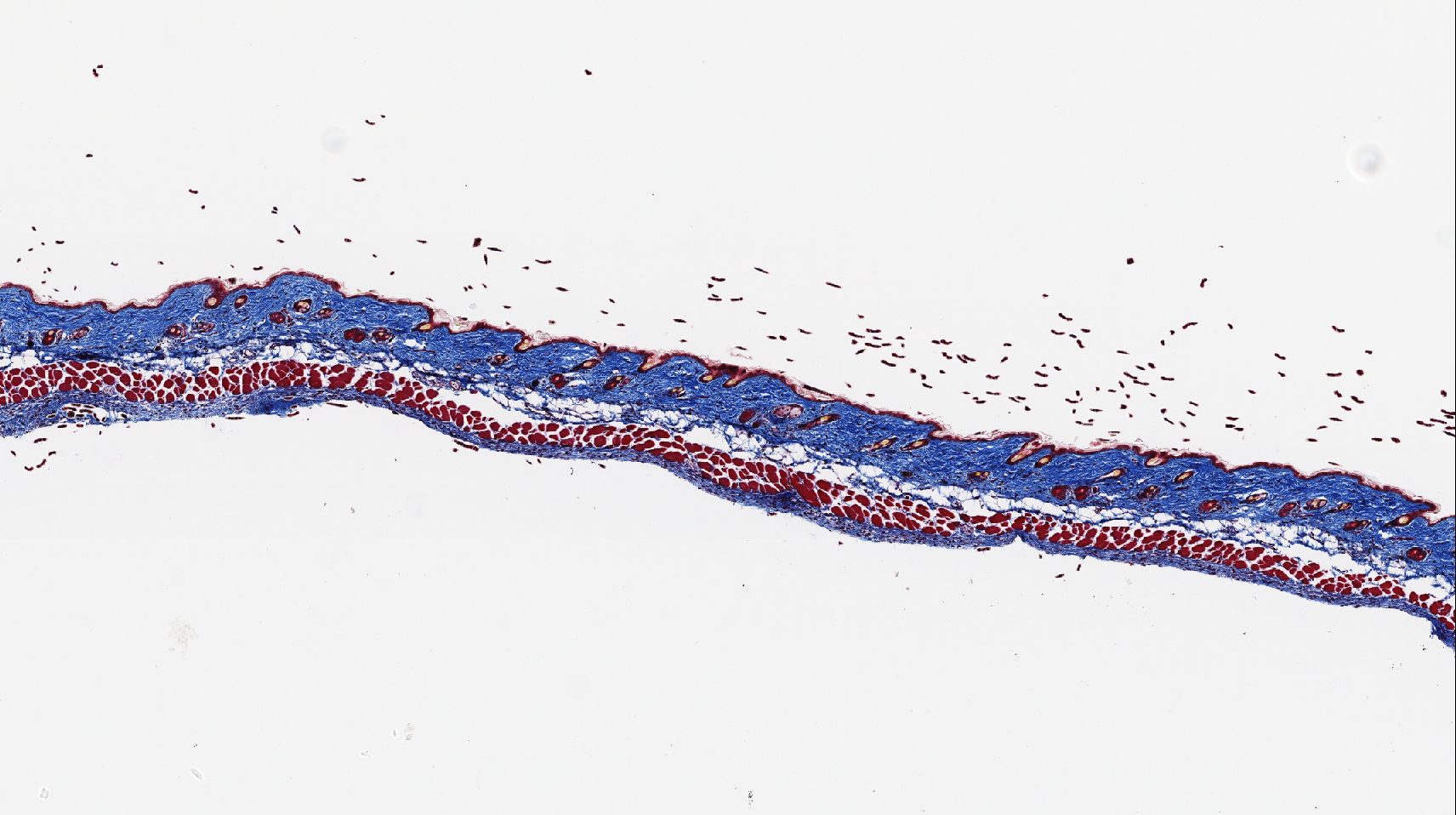

Supplement: S1 File — Images from dorsal skin Masson Trichrome stainings. Three male and three female mice per genotype were used. ASPN WT are wild-type, ASPN KO are Aspn-/-. (ZIP) [file pone.0184028.s004.zip › S1 File/ASPN KO Female-1 Overview.tif]

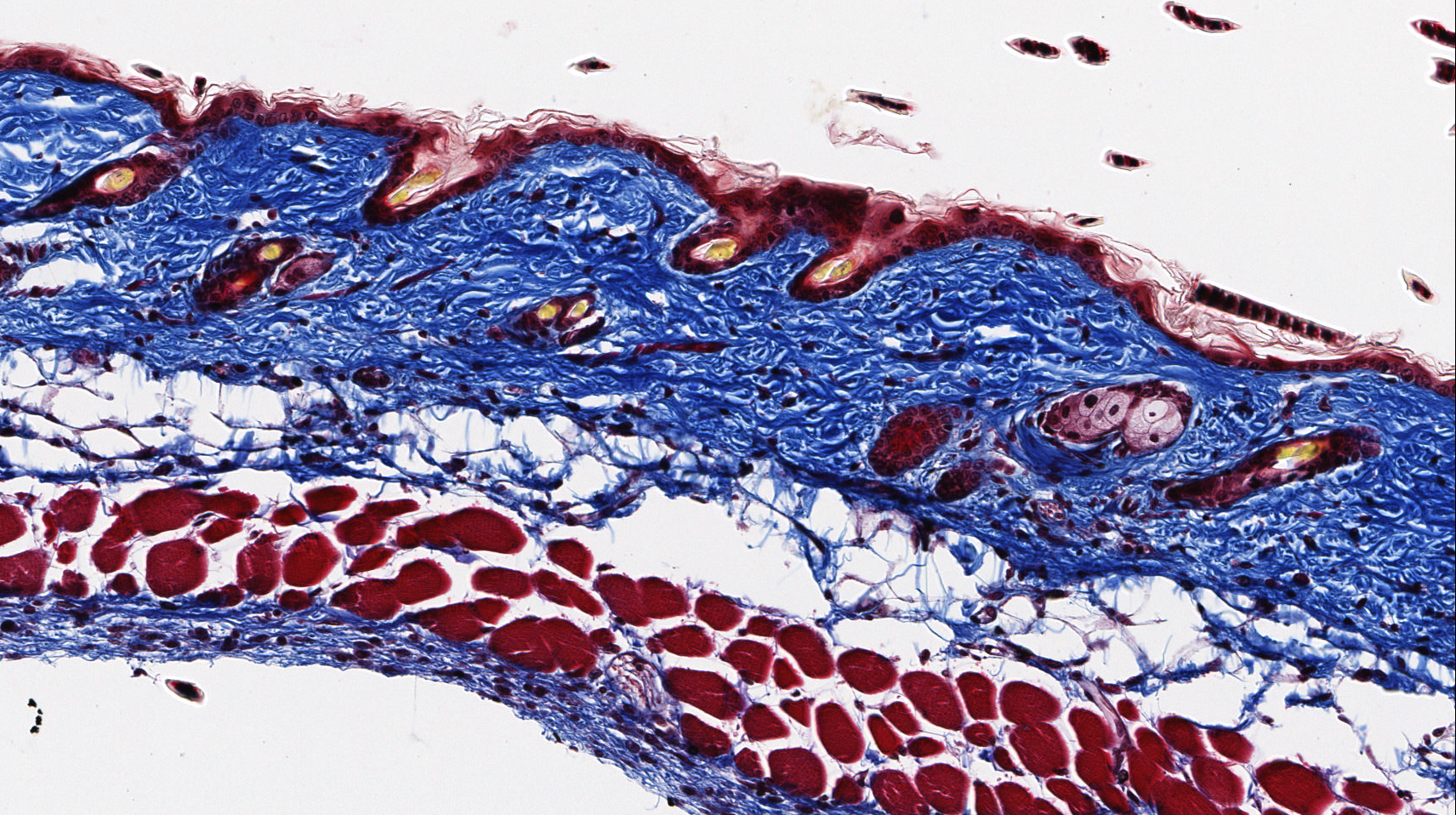

Supplement: S1 File — Images from dorsal skin Masson Trichrome stainings. Three male and three female mice per genotype were used. ASPN WT are wild-type, ASPN KO are Aspn-/-. (ZIP) [file pone.0184028.s004.zip › S1 File/ASPN KO Female-1 Zoom.tif]

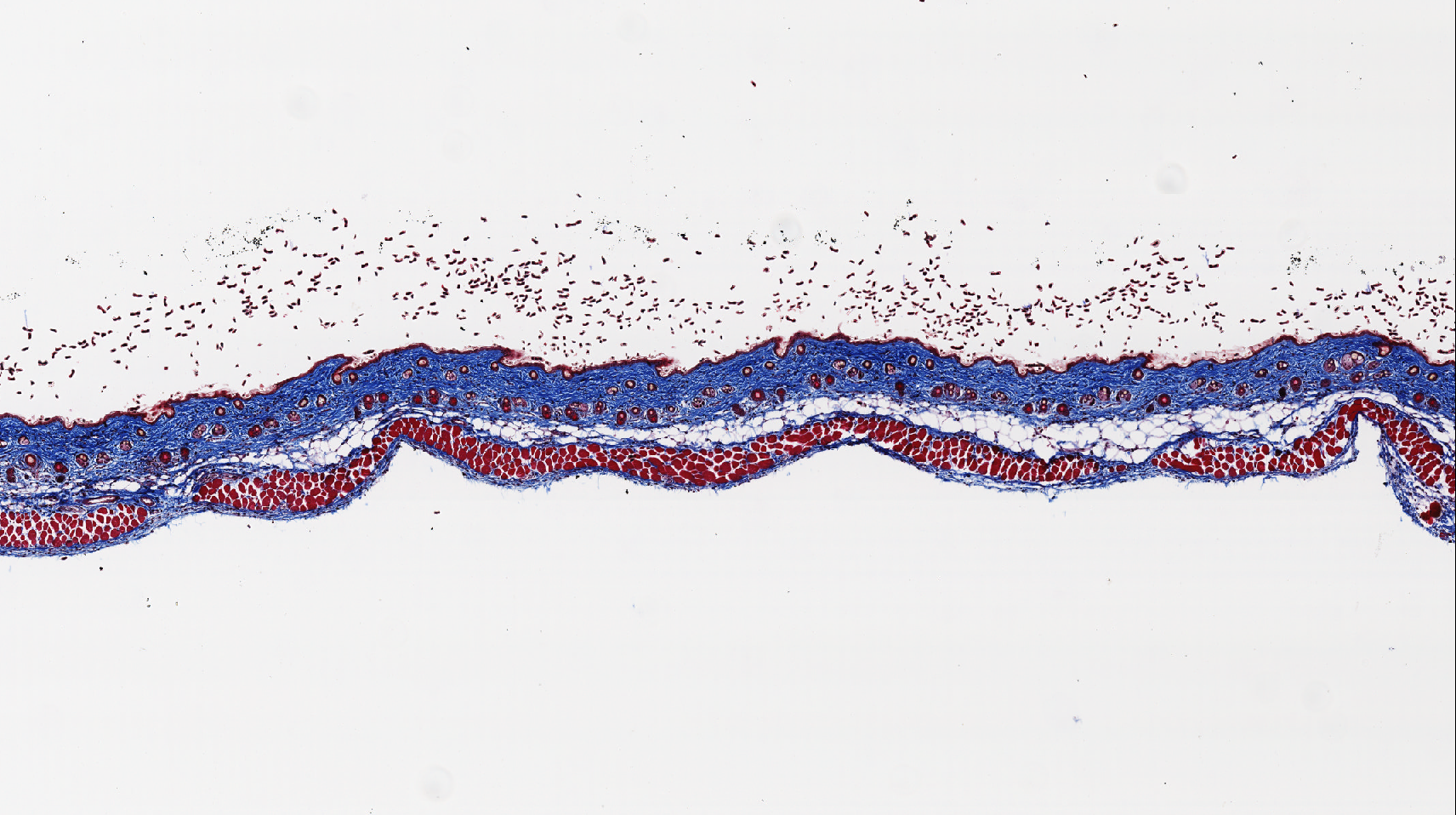

Supplement: S1 File — Images from dorsal skin Masson Trichrome stainings. Three male and three female mice per genotype were used. ASPN WT are wild-type, ASPN KO are Aspn-/-. (ZIP) [file pone.0184028.s004.zip › S1 File/ASPN KO Female-2 Overview.tif]

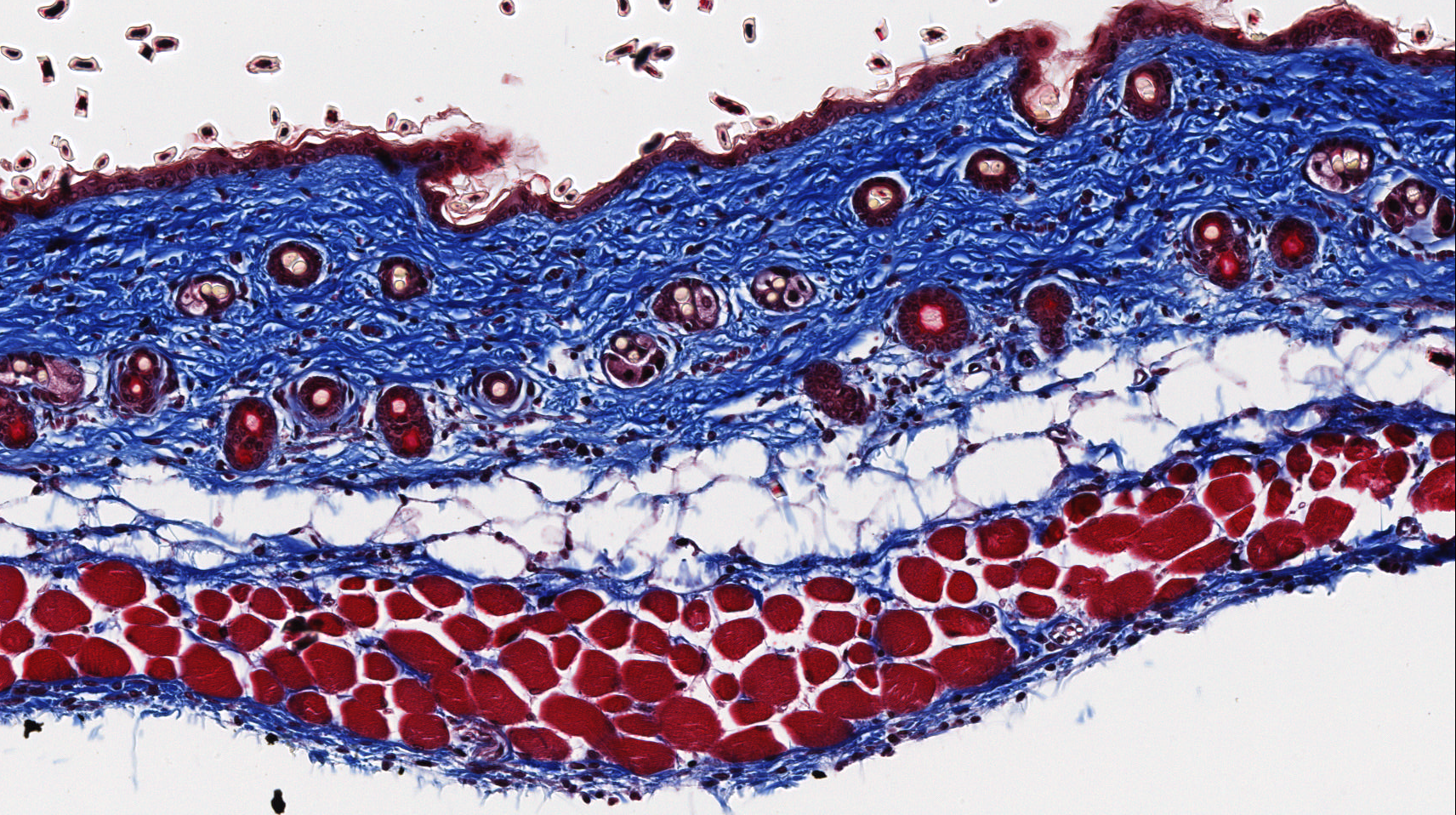

Supplement: S1 File — Images from dorsal skin Masson Trichrome stainings. Three male and three female mice per genotype were used. ASPN WT are wild-type, ASPN KO are Aspn-/-. (ZIP) [file pone.0184028.s004.zip › S1 File/ASPN KO Female-2 Zoom.tif]

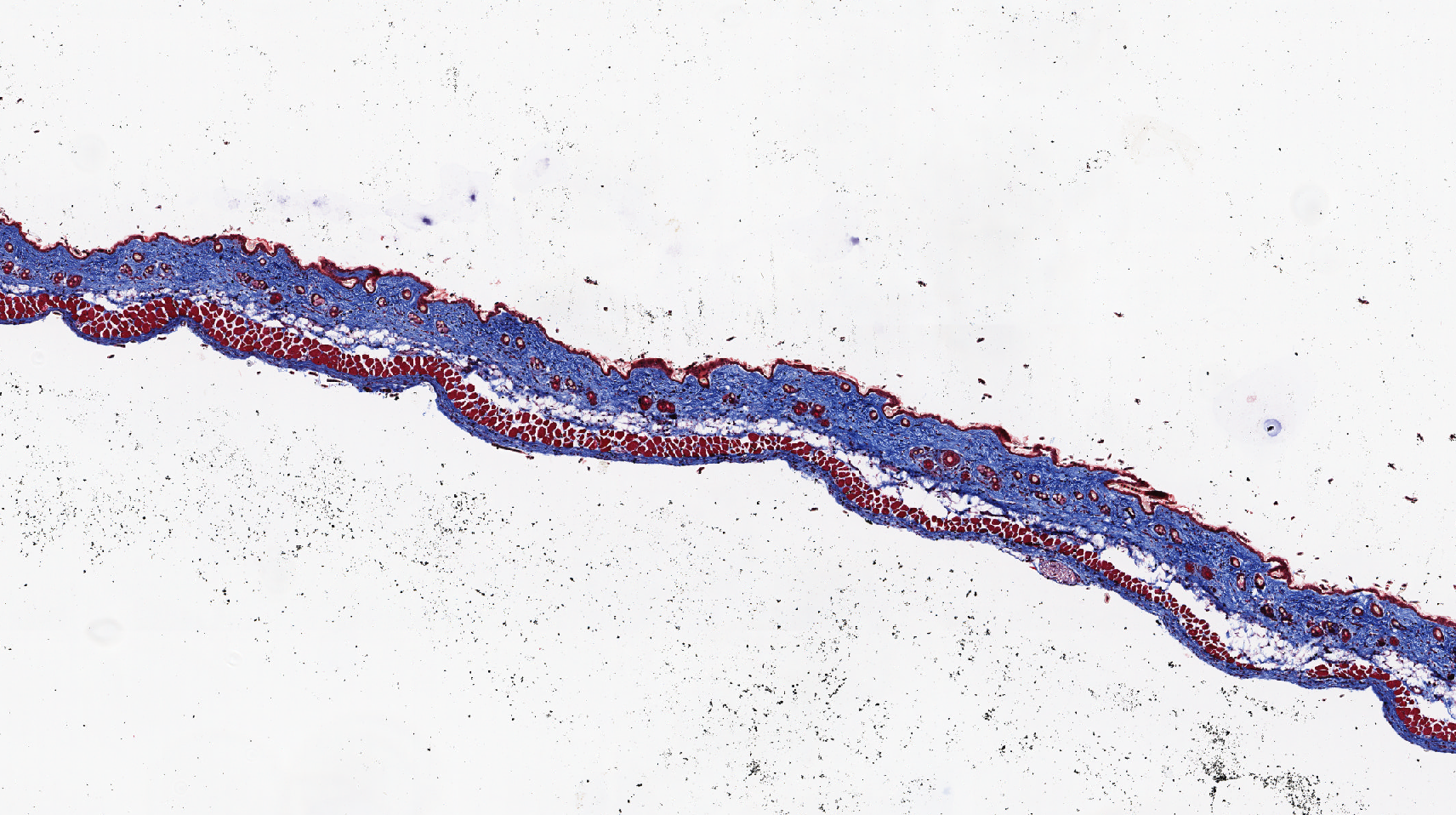

Supplement: S1 File — Images from dorsal skin Masson Trichrome stainings. Three male and three female mice per genotype were used. ASPN WT are wild-type, ASPN KO are Aspn-/-. (ZIP) [file pone.0184028.s004.zip › S1 File/ASPN KO Female-3 Overview.tif]

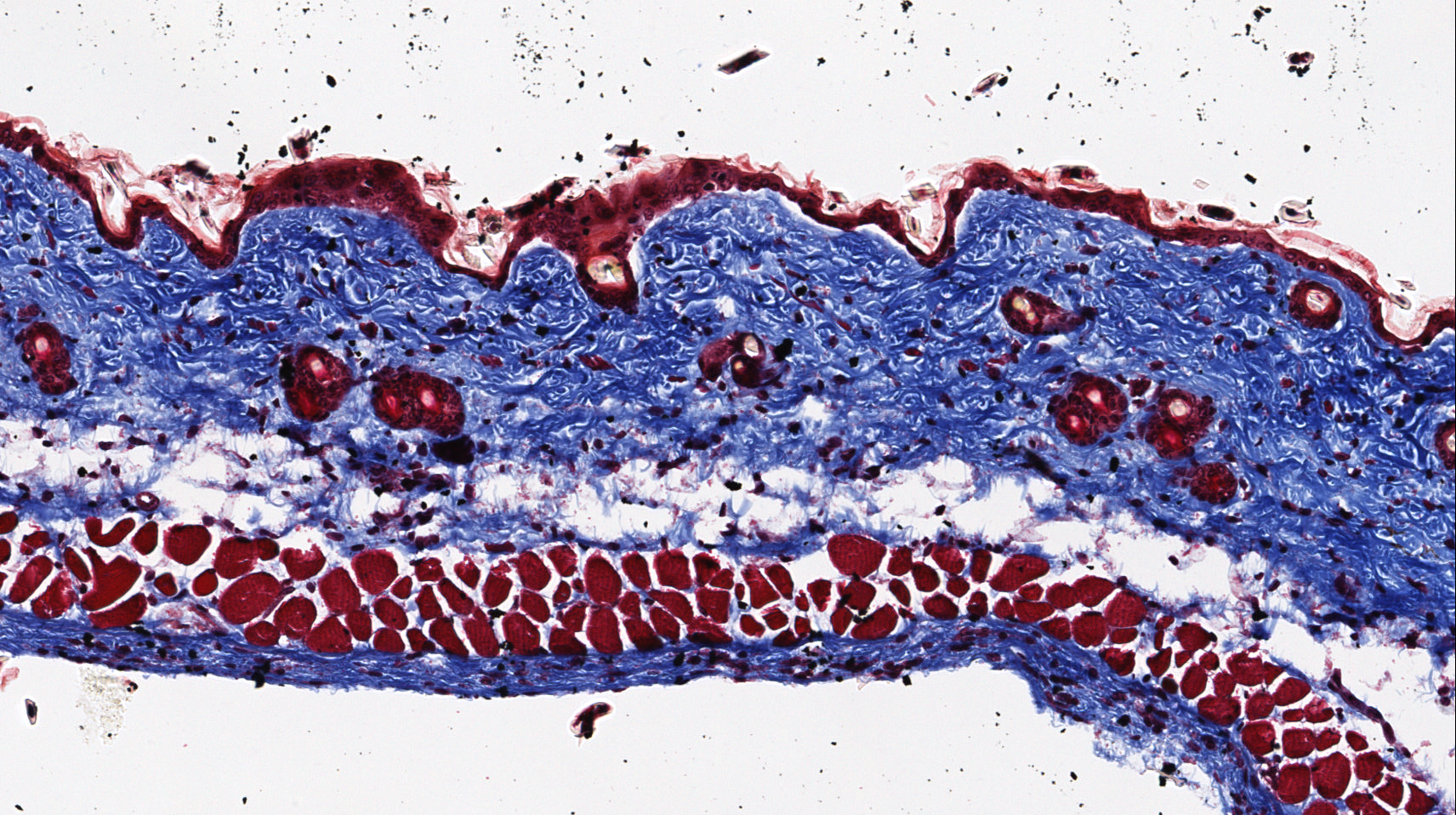

Supplement: S1 File — Images from dorsal skin Masson Trichrome stainings. Three male and three female mice per genotype were used. ASPN WT are wild-type, ASPN KO are Aspn-/-. (ZIP) [file pone.0184028.s004.zip › S1 File/ASPN KO Female-3 Zoom.tif]

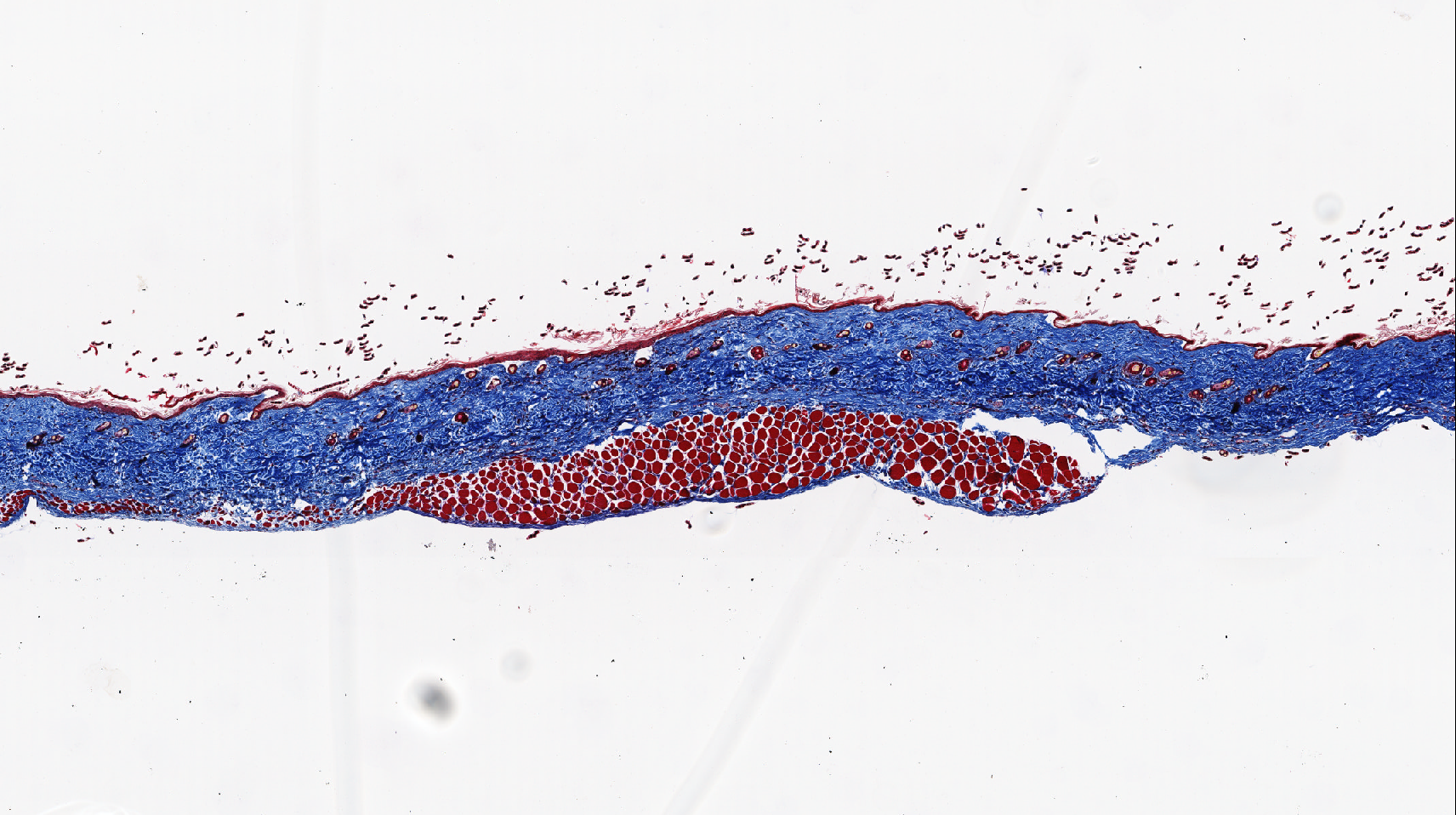

Supplement: S1 File — Images from dorsal skin Masson Trichrome stainings. Three male and three female mice per genotype were used. ASPN WT are wild-type, ASPN KO are Aspn-/-. (ZIP) [file pone.0184028.s004.zip › S1 File/ASPN KO Male-1 Overview.tif]

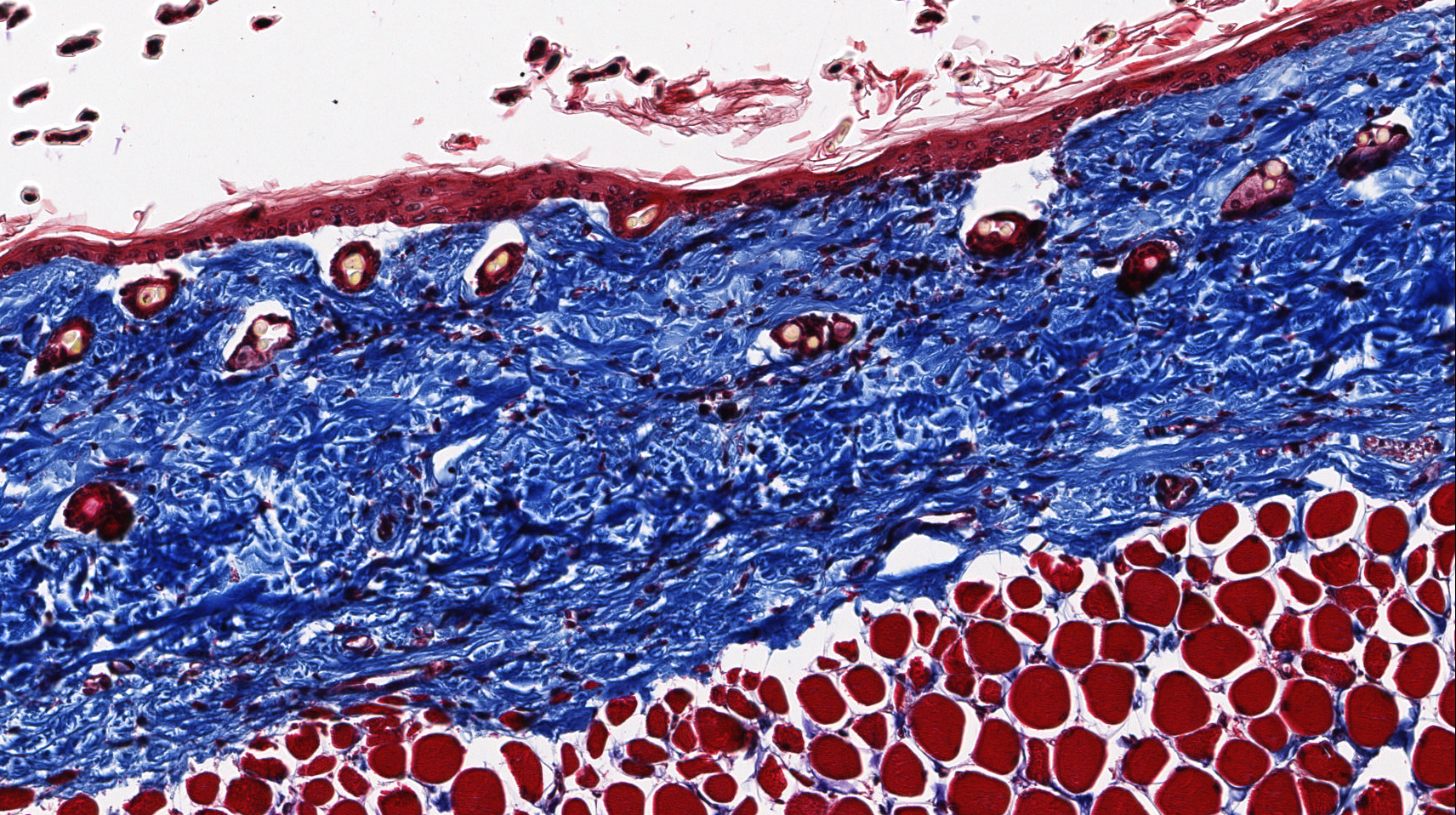

Supplement: S1 File — Images from dorsal skin Masson Trichrome stainings. Three male and three female mice per genotype were used. ASPN WT are wild-type, ASPN KO are Aspn-/-. (ZIP) [file pone.0184028.s004.zip › S1 File/ASPN KO Male-1 Zoom.tif]

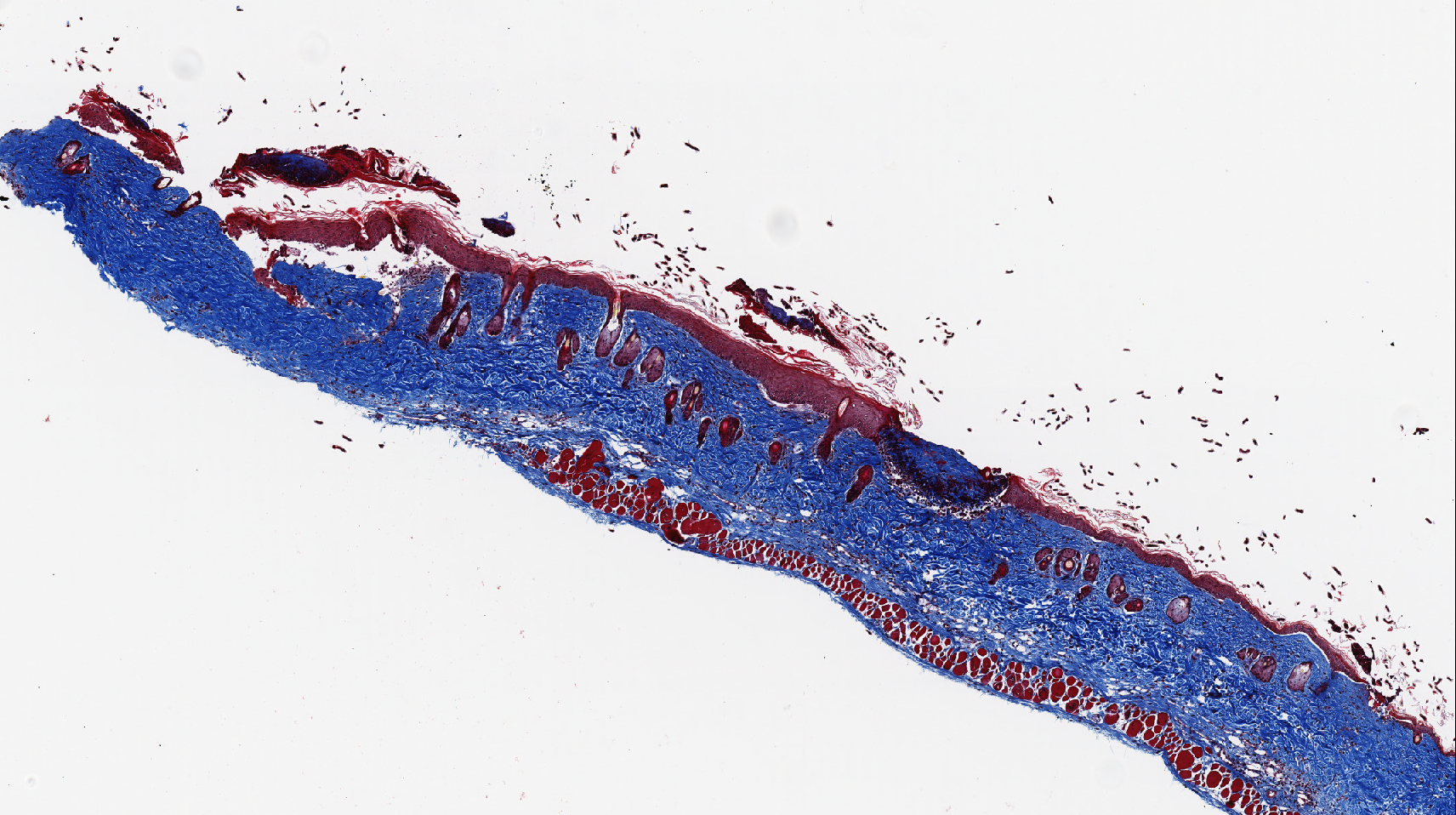

Supplement: S1 File — Images from dorsal skin Masson Trichrome stainings. Three male and three female mice per genotype were used. ASPN WT are wild-type, ASPN KO are Aspn-/-. (ZIP) [file pone.0184028.s004.zip › S1 File/ASPN KO Male-2 Overview.tif]

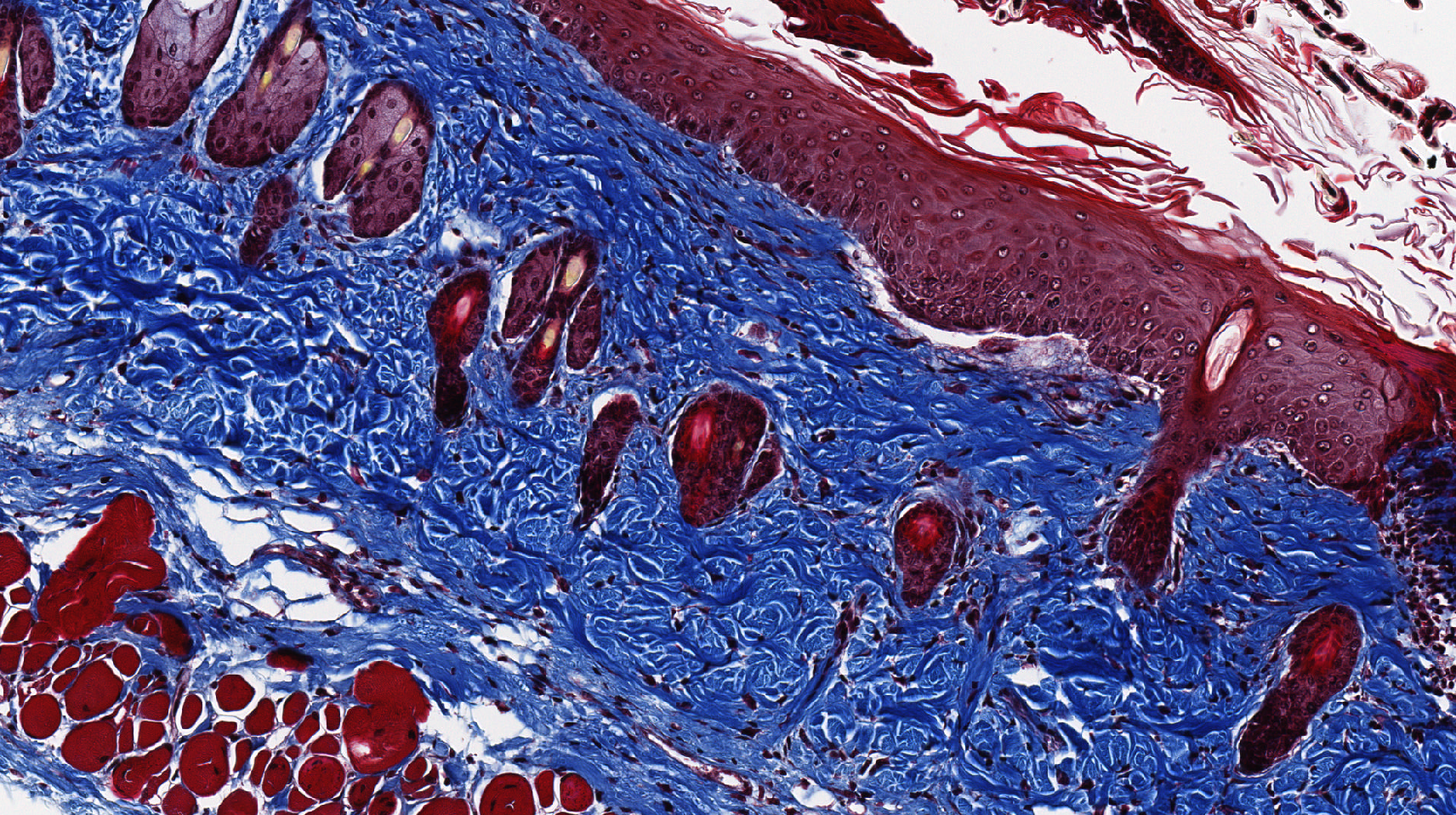

Supplement: S1 File — Images from dorsal skin Masson Trichrome stainings. Three male and three female mice per genotype were used. ASPN WT are wild-type, ASPN KO are Aspn-/-. (ZIP) [file pone.0184028.s004.zip › S1 File/ASPN KO Male-2 Zoom.tif]

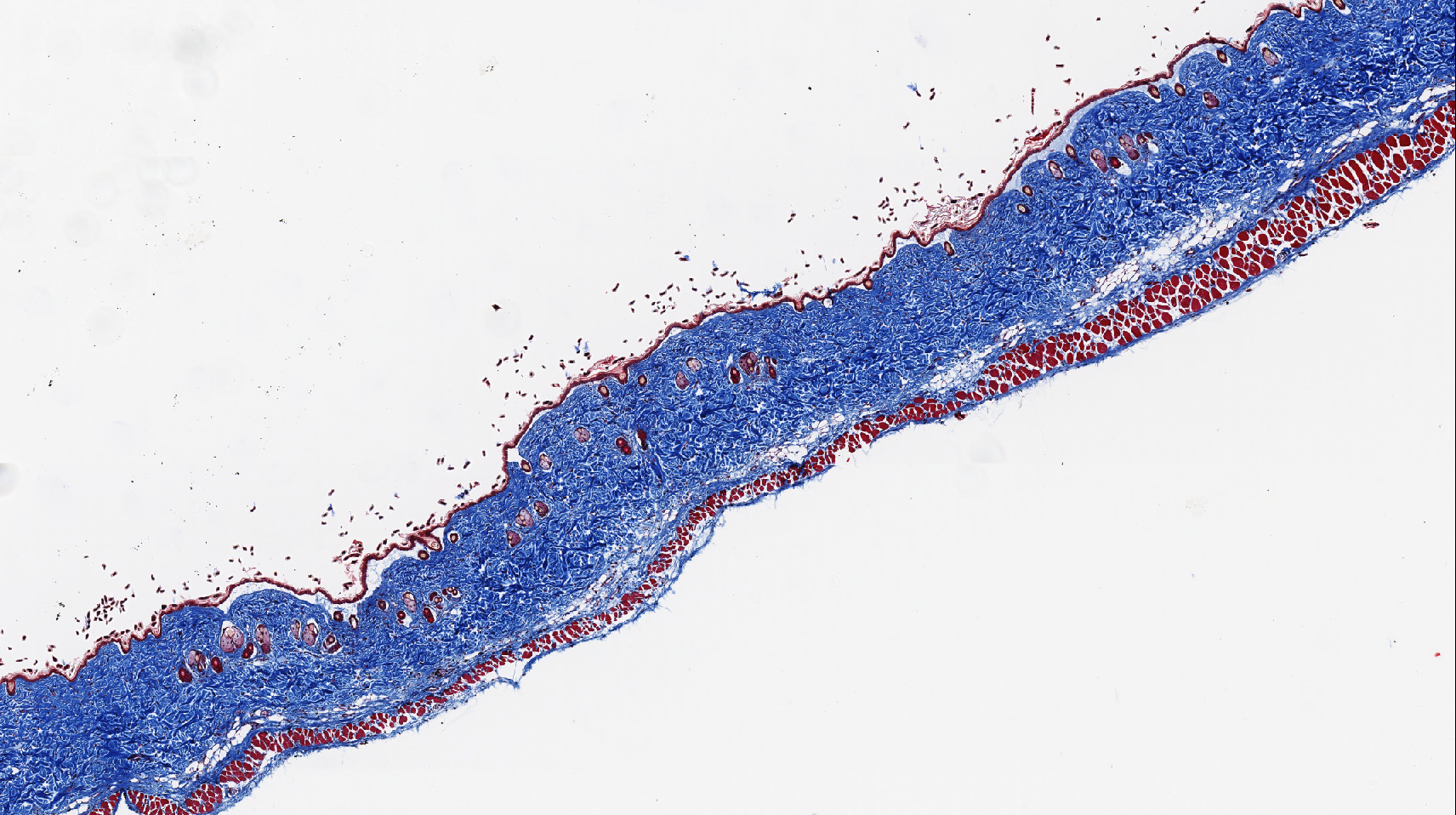

Supplement: S1 File — Images from dorsal skin Masson Trichrome stainings. Three male and three female mice per genotype were used. ASPN WT are wild-type, ASPN KO are Aspn-/-. (ZIP) [file pone.0184028.s004.zip › S1 File/ASPN KO Male-3 Overview.tif]

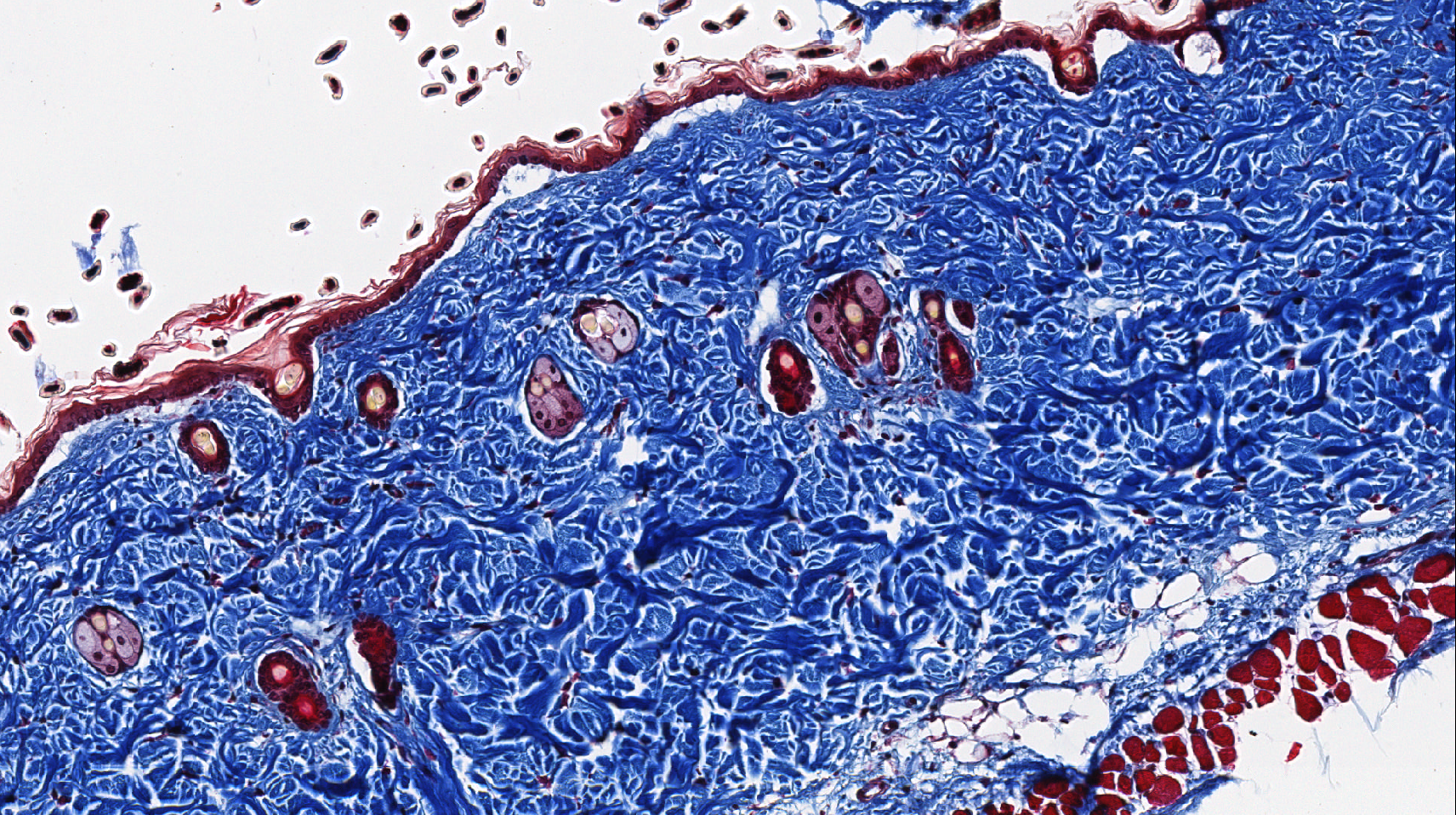

Supplement: S1 File — Images from dorsal skin Masson Trichrome stainings. Three male and three female mice per genotype were used. ASPN WT are wild-type, ASPN KO are Aspn-/-. (ZIP) [file pone.0184028.s004.zip › S1 File/ASPN KO Male-3 Zoom.tif]

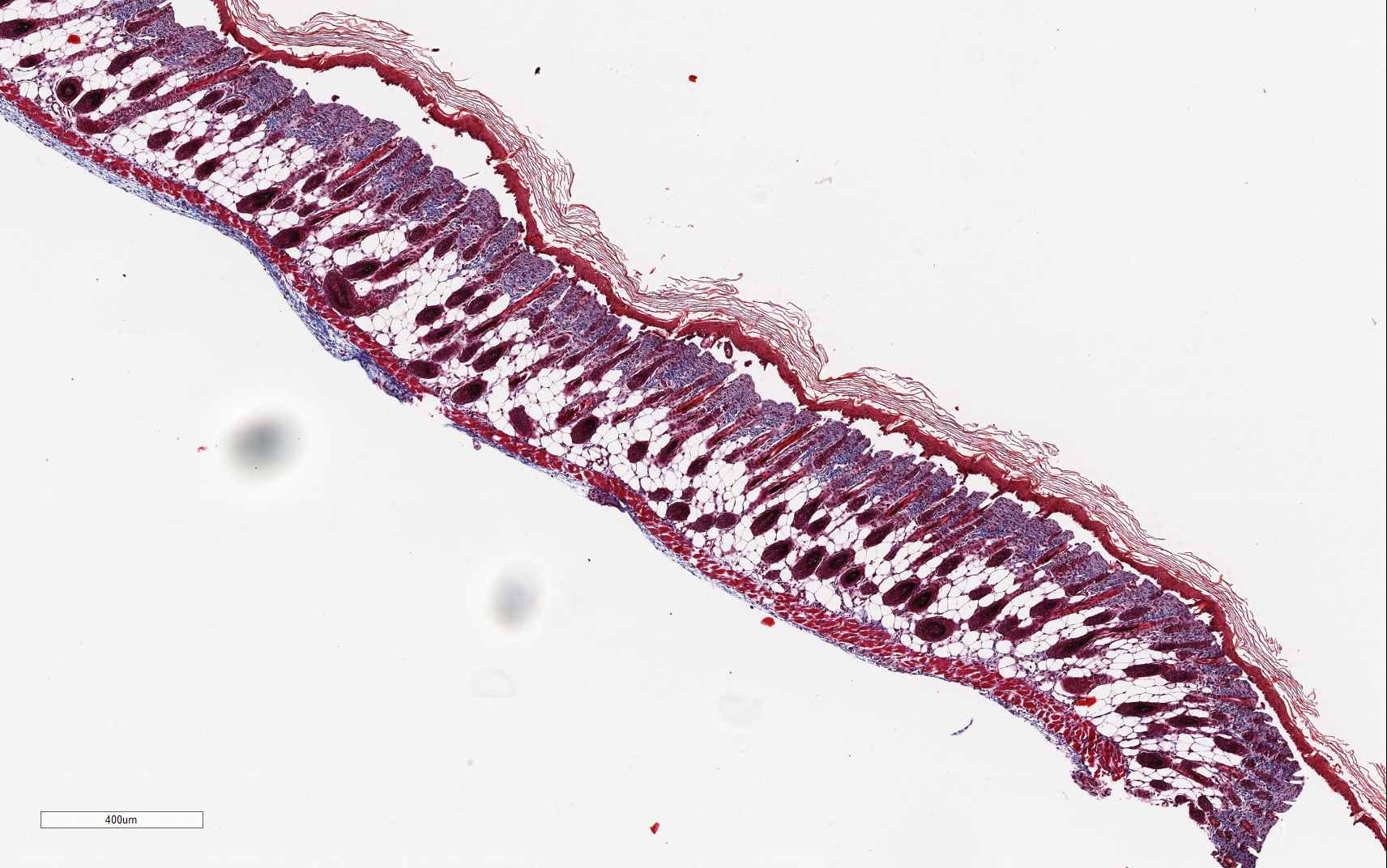

Supplement: S1 File — Images from dorsal skin Masson Trichrome stainings. Three male and three female mice per genotype were used. ASPN WT are wild-type, ASPN KO are Aspn-/-. (ZIP) [file pone.0184028.s004.zip › S1 File/ASPN WT (3) MT 5X.tif]

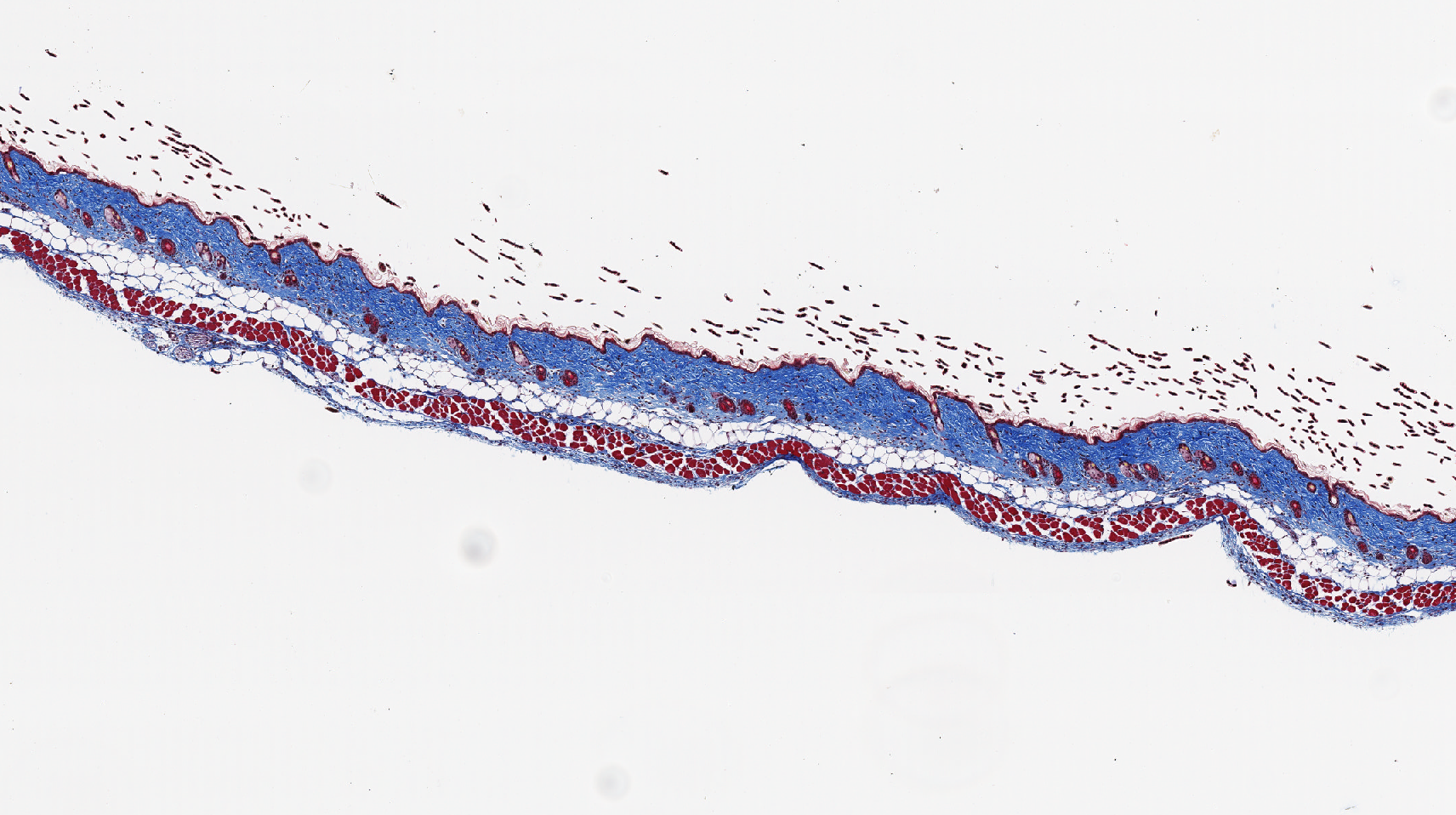

Supplement: S1 File — Images from dorsal skin Masson Trichrome stainings. Three male and three female mice per genotype were used. ASPN WT are wild-type, ASPN KO are Aspn-/-. (ZIP) [file pone.0184028.s004.zip › S1 File/ASPN WT Female-1 Overview.tif]

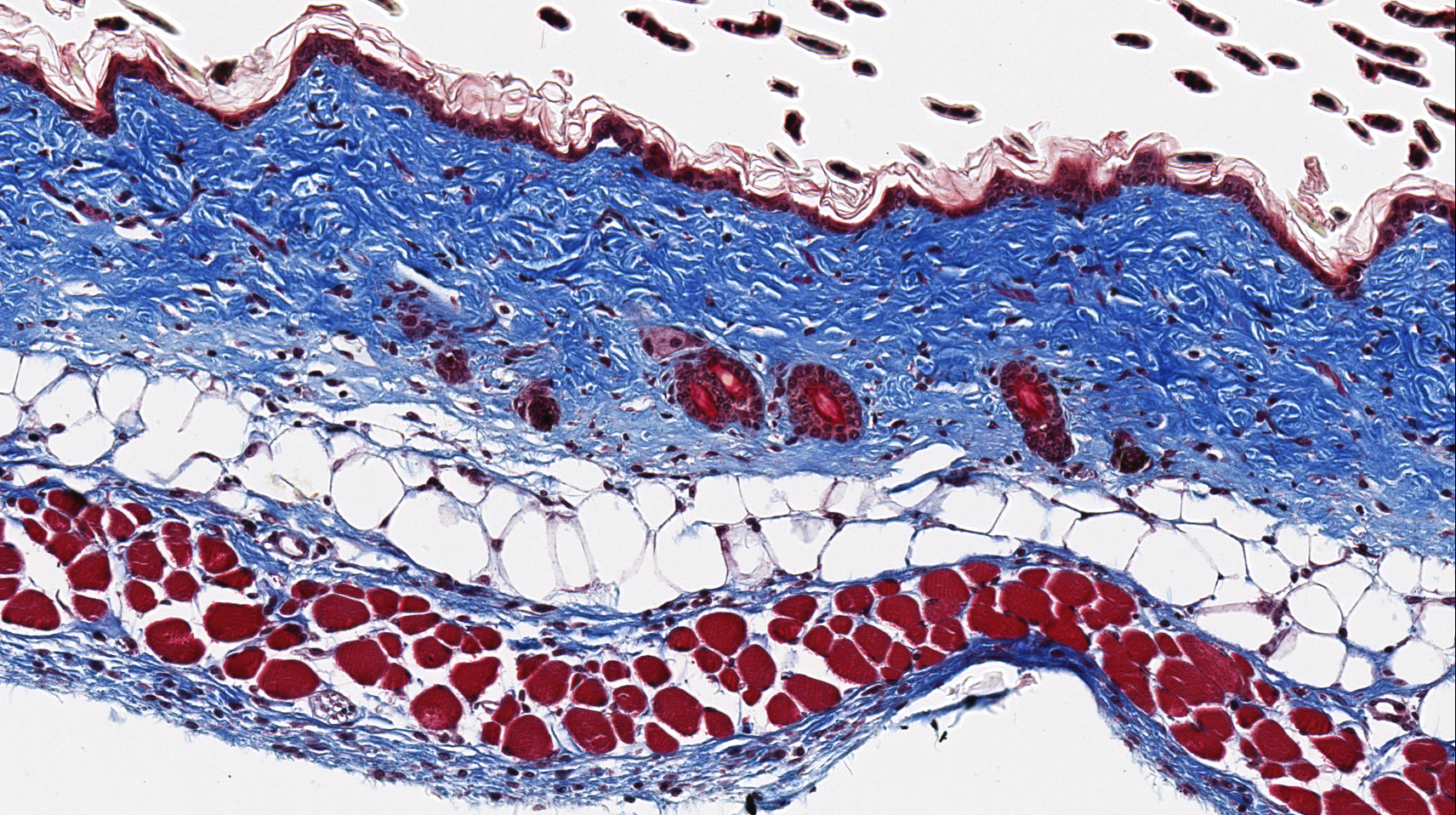

Supplement: S1 File — Images from dorsal skin Masson Trichrome stainings. Three male and three female mice per genotype were used. ASPN WT are wild-type, ASPN KO are Aspn-/-. (ZIP) [file pone.0184028.s004.zip › S1 File/ASPN WT Female-1 Zoom.tif]

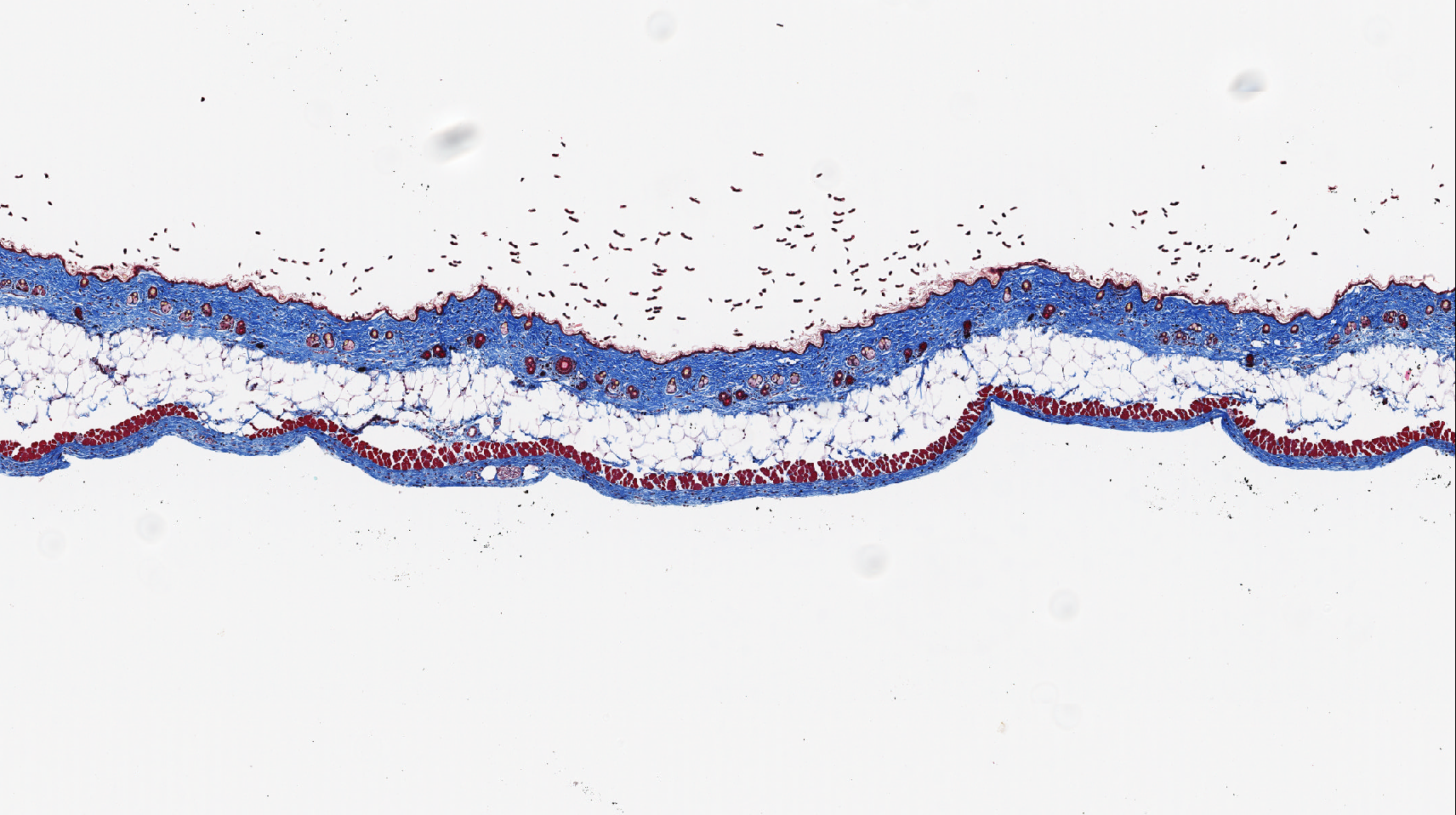

Supplement: S1 File — Images from dorsal skin Masson Trichrome stainings. Three male and three female mice per genotype were used. ASPN WT are wild-type, ASPN KO are Aspn-/-. (ZIP) [file pone.0184028.s004.zip › S1 File/ASPN WT Female-2 Overview.tif]

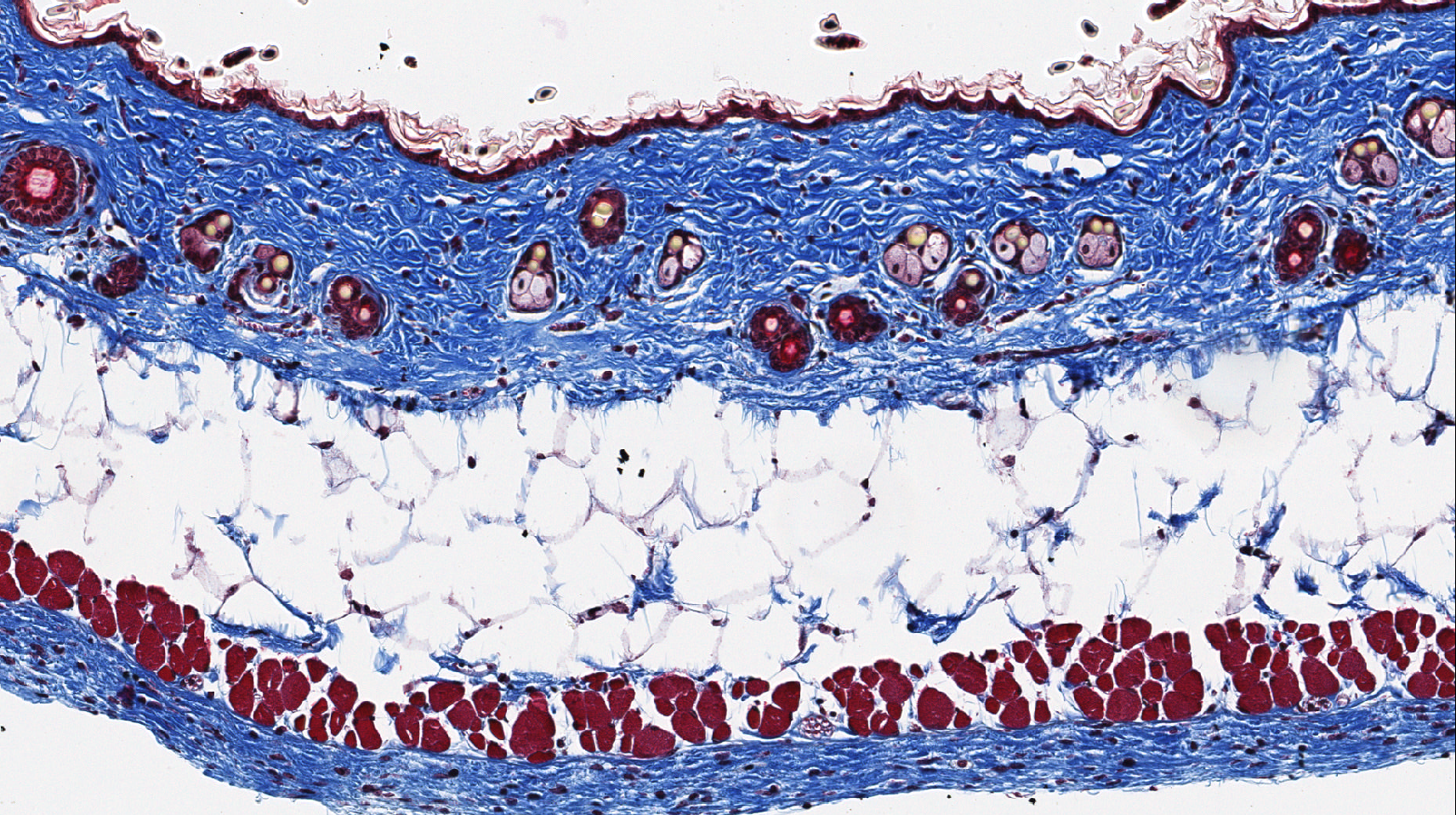

Supplement: S1 File — Images from dorsal skin Masson Trichrome stainings. Three male and three female mice per genotype were used. ASPN WT are wild-type, ASPN KO are Aspn-/-. (ZIP) [file pone.0184028.s004.zip › S1 File/ASPN WT Female-2 Zoom.tif]

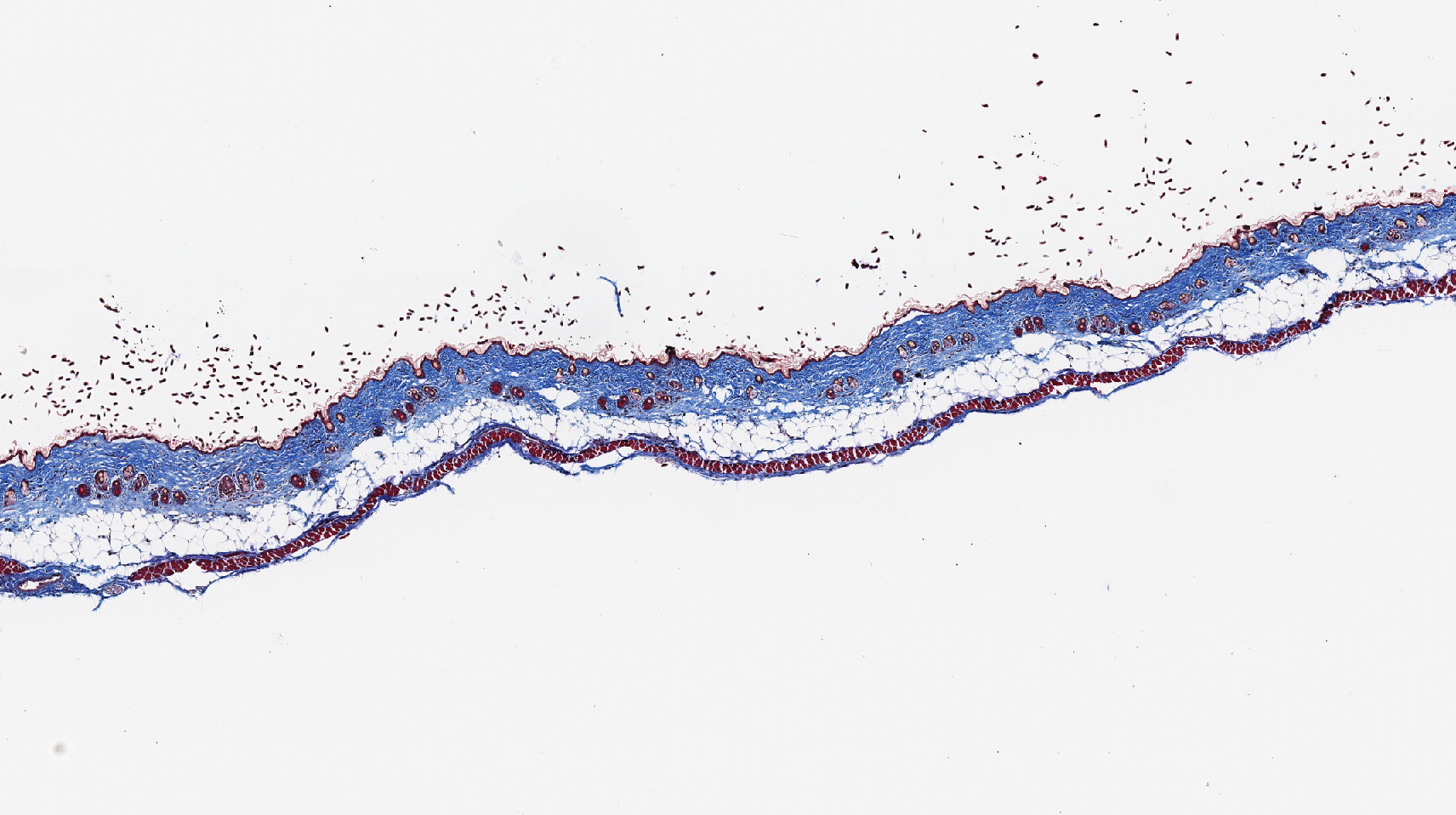

Supplement: S1 File — Images from dorsal skin Masson Trichrome stainings. Three male and three female mice per genotype were used. ASPN WT are wild-type, ASPN KO are Aspn-/-. (ZIP) [file pone.0184028.s004.zip › S1 File/ASPN WT Female-3 Overview.tif]

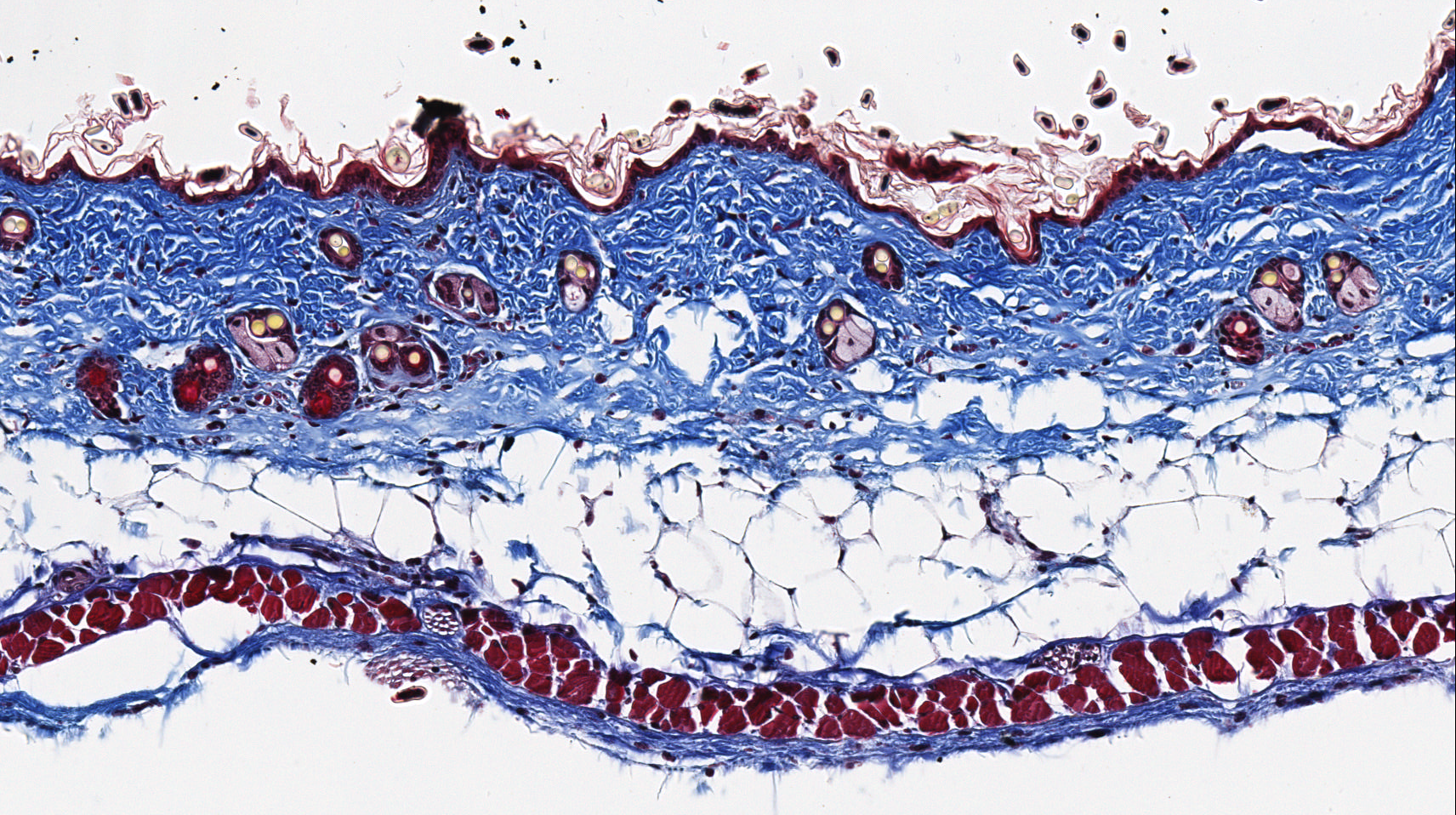

Supplement: S1 File — Images from dorsal skin Masson Trichrome stainings. Three male and three female mice per genotype were used. ASPN WT are wild-type, ASPN KO are Aspn-/-. (ZIP) [file pone.0184028.s004.zip › S1 File/ASPN WT Female-3 Zoom.tif]

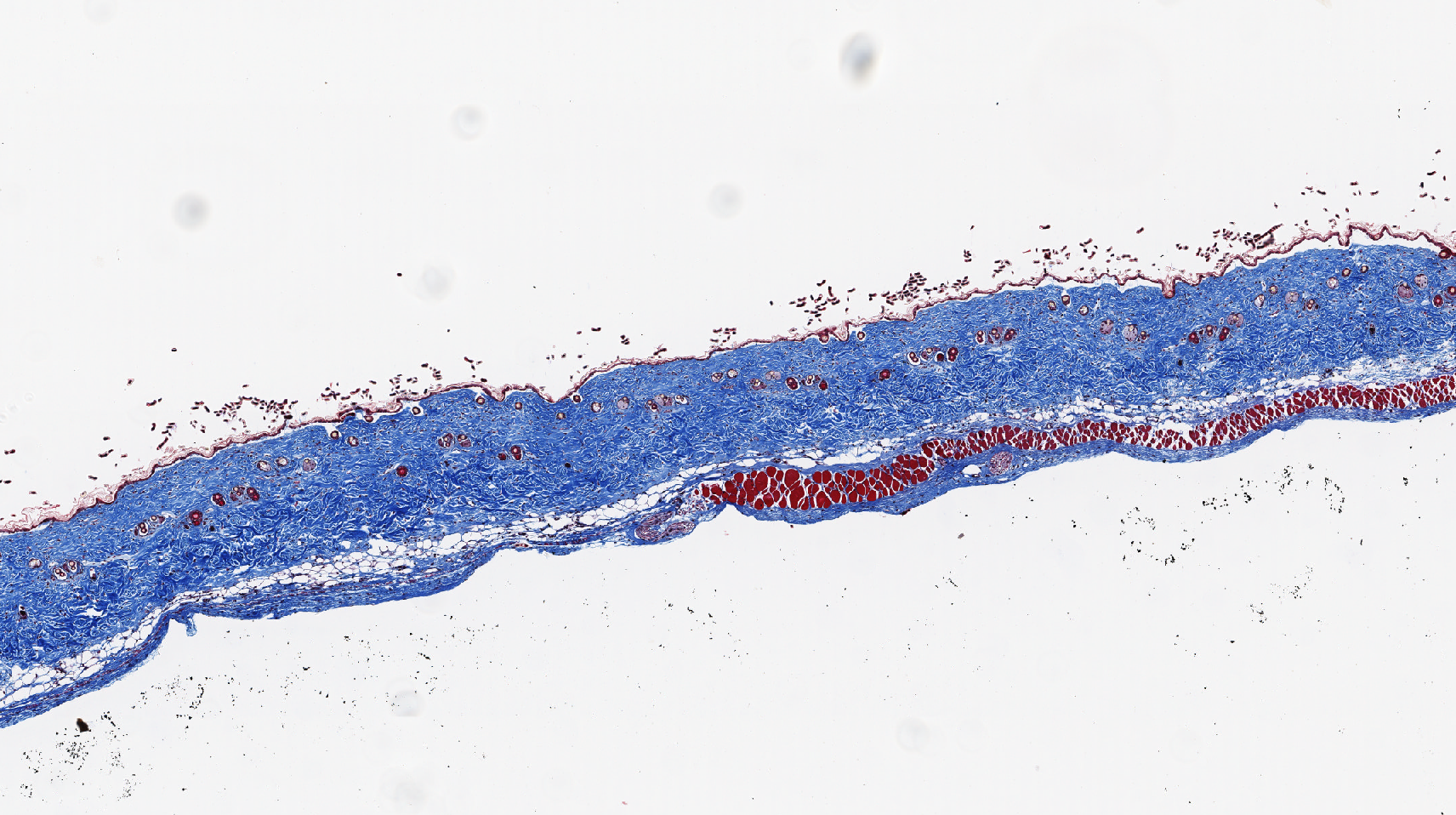

Supplement: S1 File — Images from dorsal skin Masson Trichrome stainings. Three male and three female mice per genotype were used. ASPN WT are wild-type, ASPN KO are Aspn-/-. (ZIP) [file pone.0184028.s004.zip › S1 File/ASPN WT Male-1 Overview.tif]

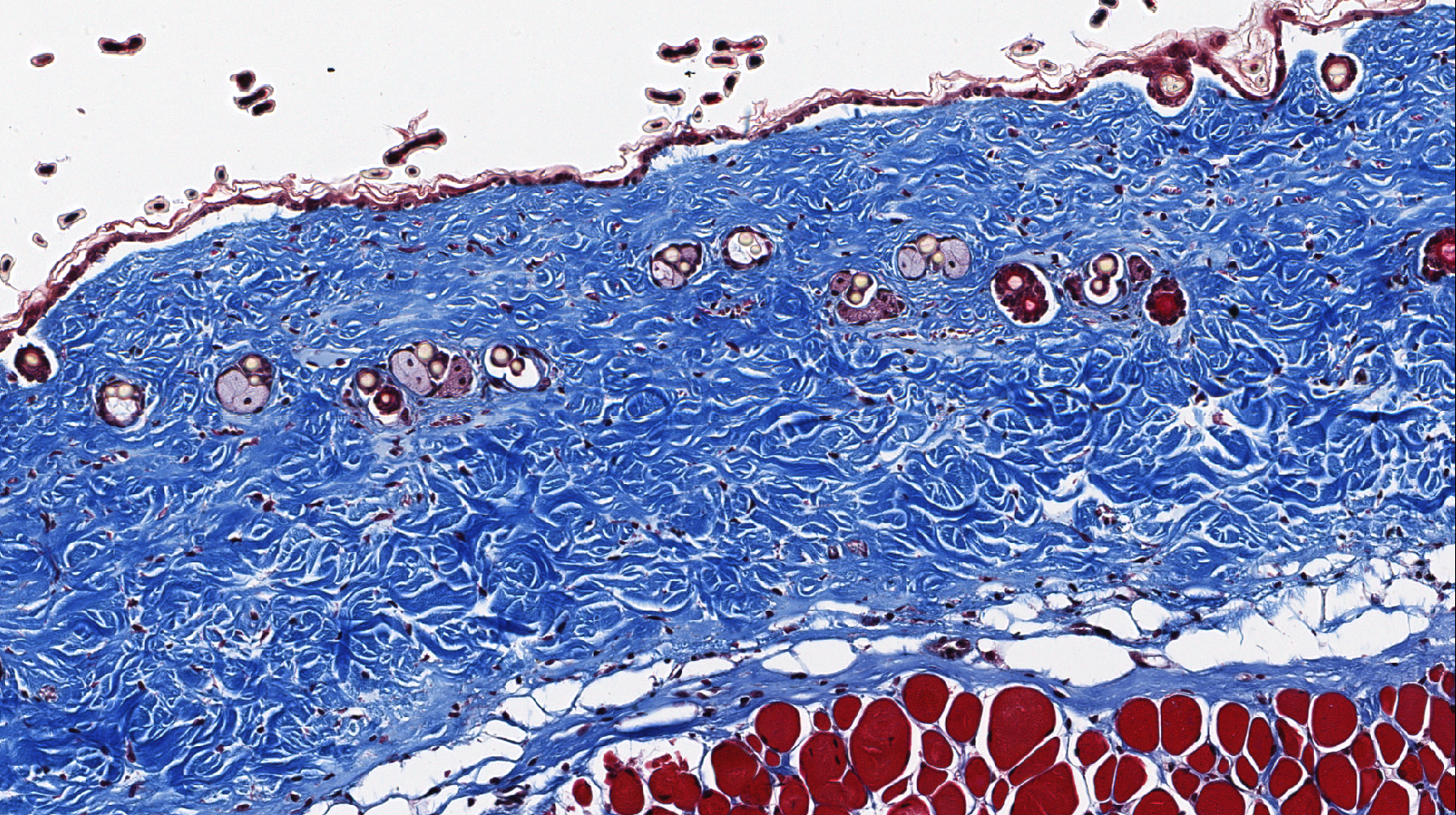

Supplement: S1 File — Images from dorsal skin Masson Trichrome stainings. Three male and three female mice per genotype were used. ASPN WT are wild-type, ASPN KO are Aspn-/-. (ZIP) [file pone.0184028.s004.zip › S1 File/ASPN WT Male-1 Zoom.tif]

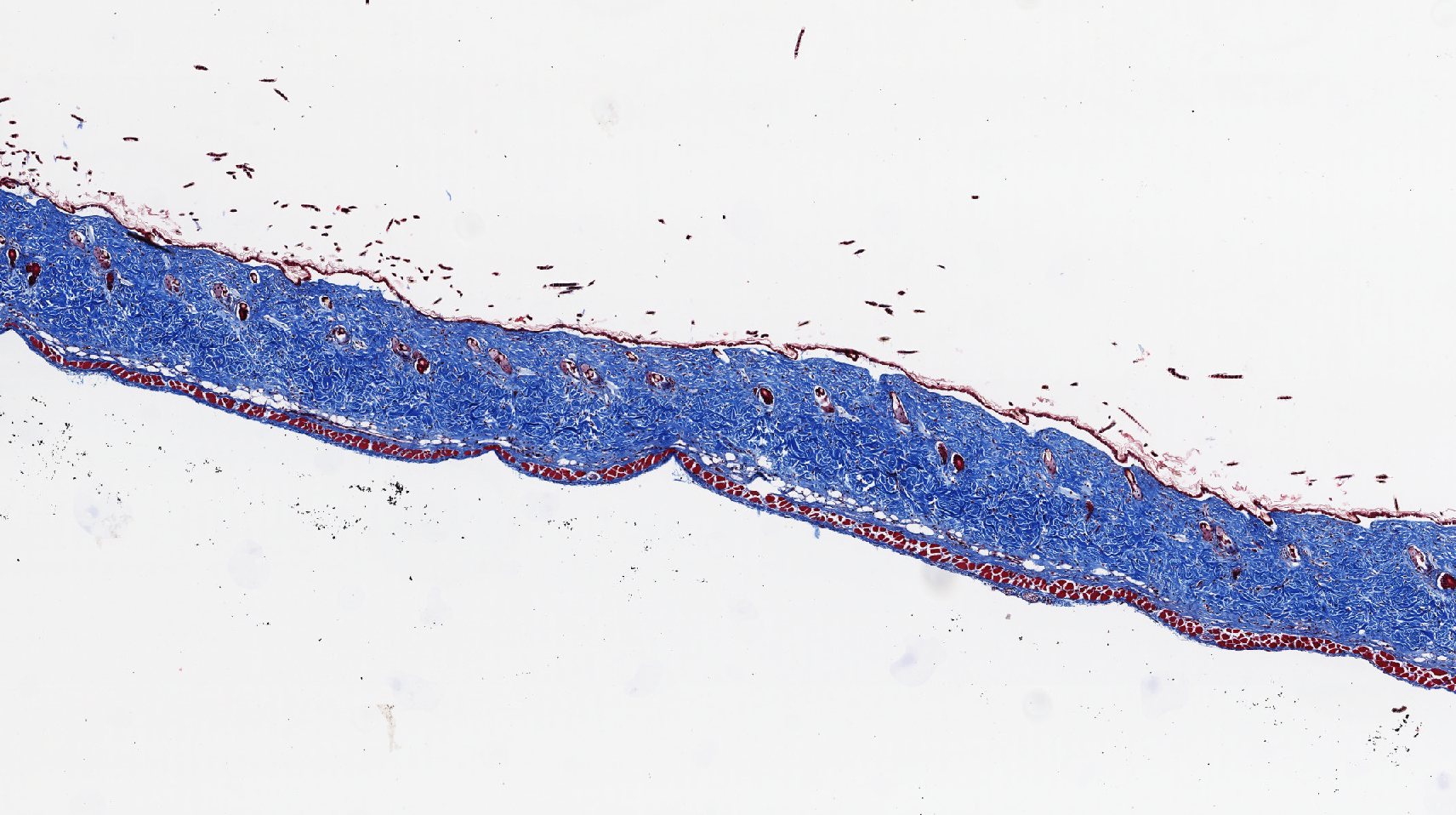

Supplement: S1 File — Images from dorsal skin Masson Trichrome stainings. Three male and three female mice per genotype were used. ASPN WT are wild-type, ASPN KO are Aspn-/-. (ZIP) [file pone.0184028.s004.zip › S1 File/ASPN WT Male-2 Overview.tif]

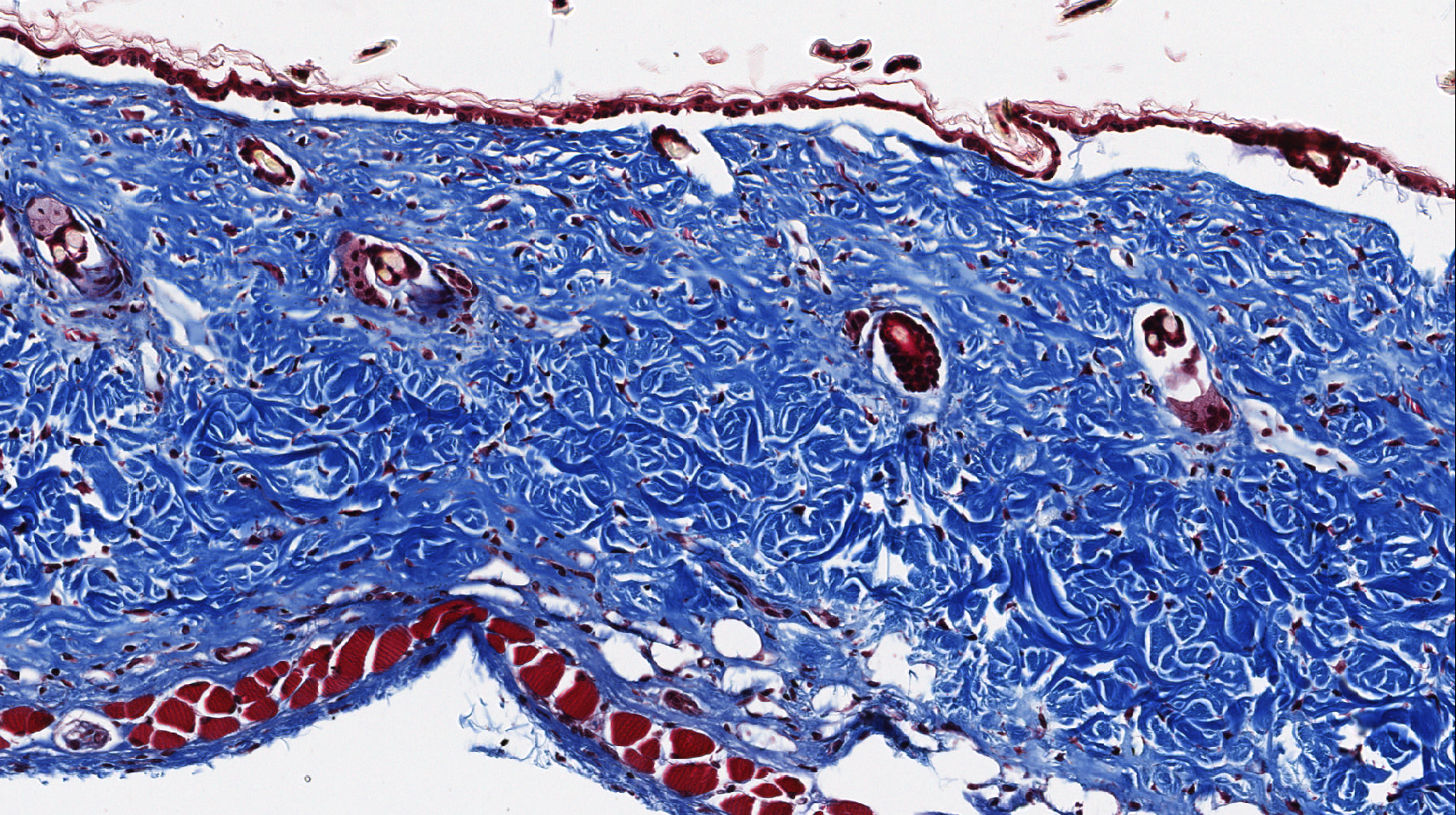

Supplement: S1 File — Images from dorsal skin Masson Trichrome stainings. Three male and three female mice per genotype were used. ASPN WT are wild-type, ASPN KO are Aspn-/-. (ZIP) [file pone.0184028.s004.zip › S1 File/ASPN WT Male-2 Zoom.tif]

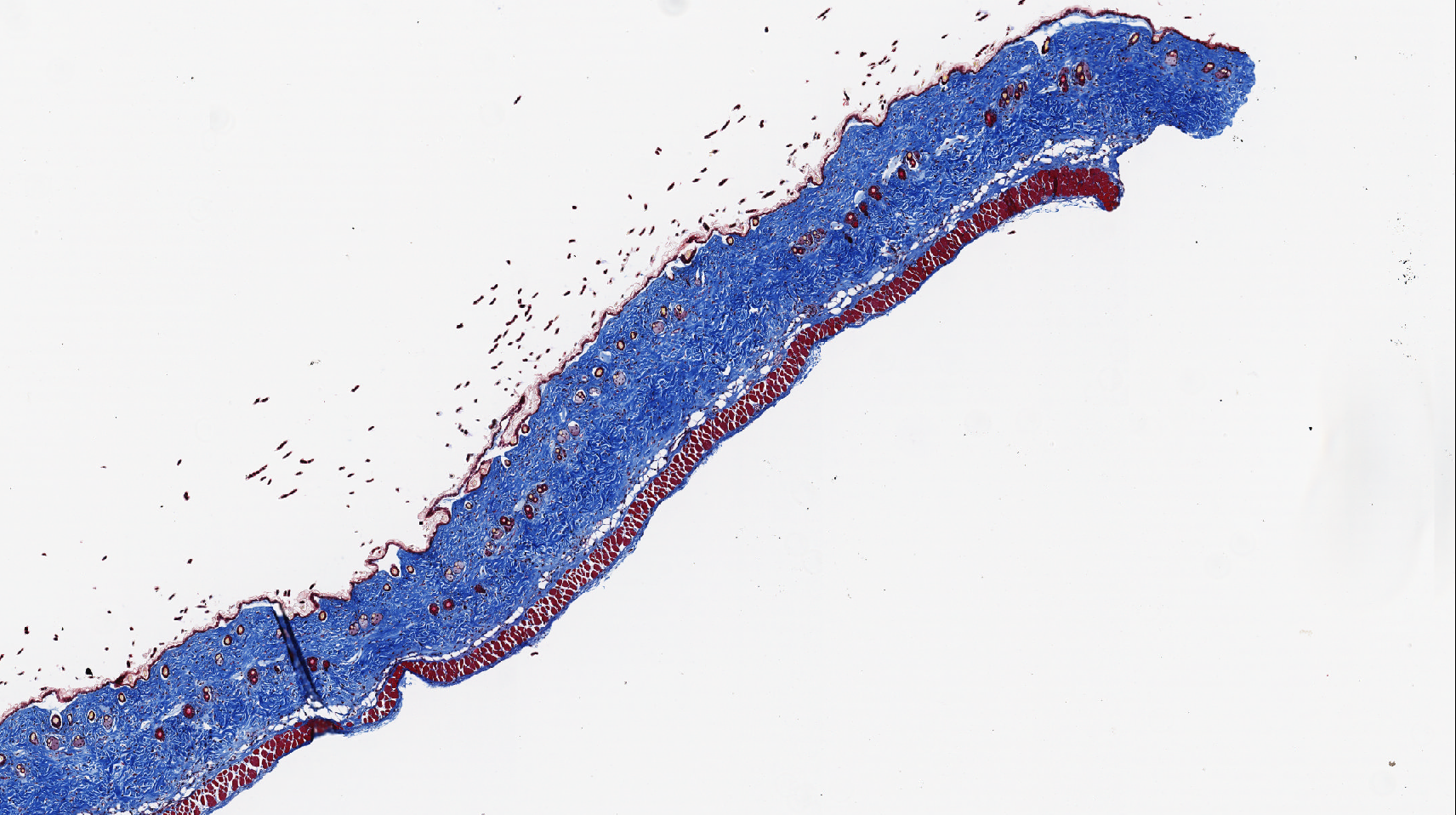

Supplement: S1 File — Images from dorsal skin Masson Trichrome stainings. Three male and three female mice per genotype were used. ASPN WT are wild-type, ASPN KO are Aspn-/-. (ZIP) [file pone.0184028.s004.zip › S1 File/ASPN WT Male-3 Overview.tif]

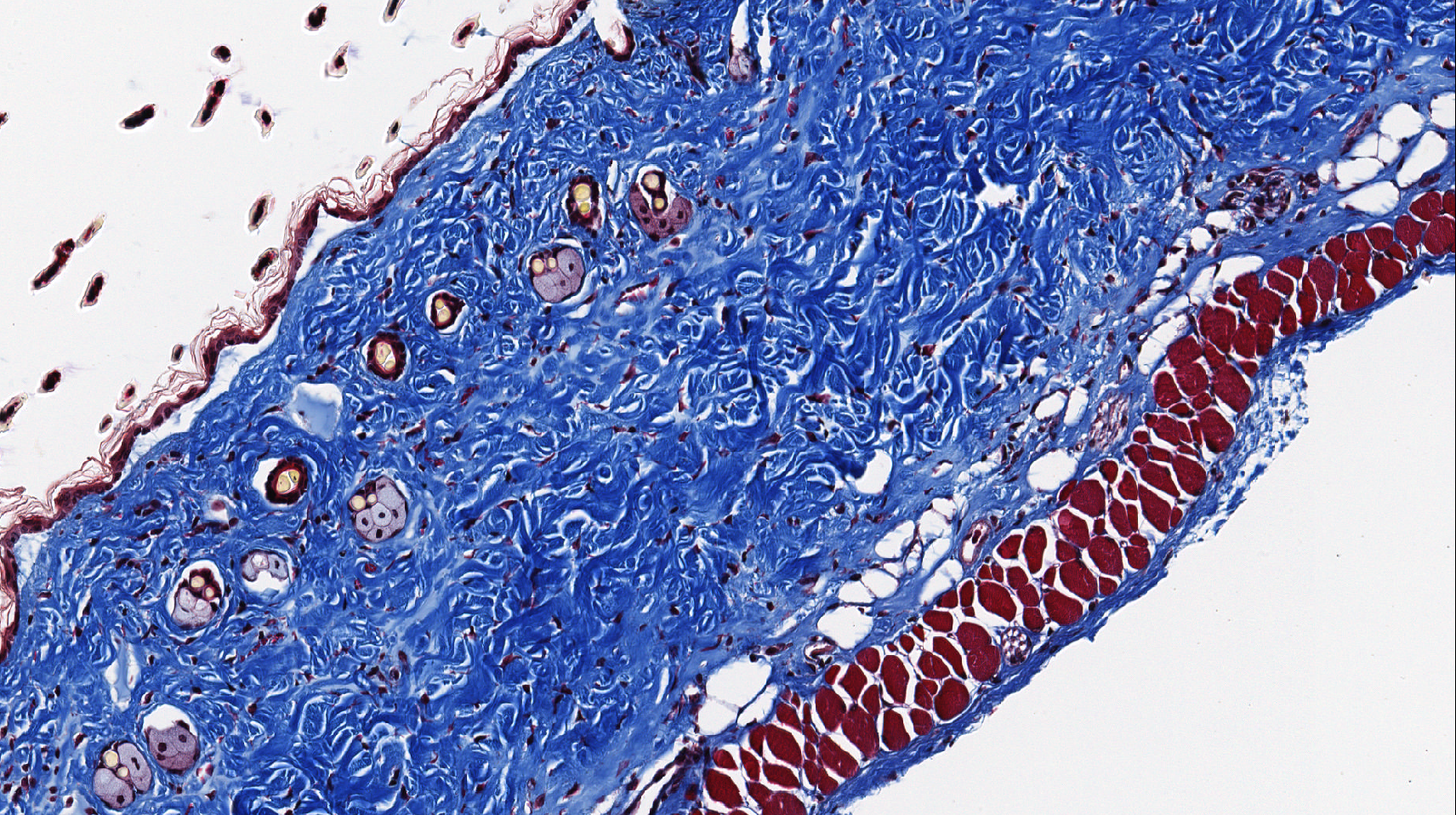

Supplement: S1 File — Images from dorsal skin Masson Trichrome stainings. Three male and three female mice per genotype were used. ASPN WT are wild-type, ASPN KO are Aspn-/-. (ZIP) [file pone.0184028.s004.zip › S1 File/ASPN WT Male-3 Zoom.tif]
