# Supplementary material for: Asporin-deficient mice have tougher skin and altered skin glycosaminoglycan content and structure
Source: PLoS One. 2017 Aug 31;12(8):e0184028. doi: 10.1371/journal.pone.0184028 (PMC5578652; doi:10.1371/journal.pone.0184028)
Supplement: S1 Table — (DOCX) [file pone.0184028.s003.docx]

| Actb | Mm00607939 |
| --- | --- |
| Col1a1 | Mm00801666 |
| Col1a2 | Mm00483888 |
| Col3a1 | Mm01254476 |
| Fmod | Mm00491215 |
| Lox | Mm00495386 |
| Loxl2 | Mm00804740 |
| Plod2 | Mm00478767 |
| Lum | Mm01248292 |
| Fbn1 | Mm00514908 |
| Mmp2 | Mm00439498 |
| Mmp3 | Mm00440295 |
| Serpinh1 | Mm00438058 |
| Mia3 | Mm00616802 |
